# Silicon flow from root to shoot in pepper: a comprehensive in silico analysis reveals a potential linkage between gene expression and hormone signaling that stimulates plant growth and metabolism

**DOI:** 10.7717/peerj.10053

**Published:** 2020-11-04

**Authors:** Fernando Carlos Gómez-Merino, Libia Iris Trejo-Téllez, Atonaltzin García-Jiménez, Hugo Fernando Escobar-Sepúlveda, Sara Monzerrat Ramírez-Olvera

**Affiliations:** 1 Department of Soil Science, Laboratory of Plant Nutrition, College of Postgraduates in Agricultural Sciences, Texcoco, State of Mexico, Mexico; 2 Department of Plant Physiology, College of Postgraduates in Agricultural Sciences, Texcoco, State of Mexico, Mexico; 3 Institute of Biological Sciences, University of Talca, Talca, Maule, Chile

**Keywords:** Solanaceae, *Capsicum annuum*, in silico analysis, Gene expression, Beneficial elements, Silicon, Hormesis

## Abstract

**Background:**

Silicon (Si) is categorized as a *quasi*-essential element for plants thanks to the benefits on growth, development and metabolism in a hormetic manner. Si uptake is cooperatively mediated by Lsi1 and Lsi2. Nevertheless, Lsi channels have not yet been identified and characterized in pepper (*Capsicum annuum*), while genes involved in major physiological processes in pepper are Si-regulated. Furthermore, Si and phytohormones may act together in regulating plant growth, metabolism and tolerance against stress. Our aim was to identify potential synergies between Si and phytohormones stimulating growth and metabolism in pepper, based on in silico data.

**Methods:**

We established a hydroponic system to test the effect of Si (0, 60, 125 and 250 mg L^−1^ Si) on the concentrations of this element in different pepper plant tissues. We also performed an in silico analysis of putative *Lsi* genes from pepper and other species, including tomato (*Solanum lycopersicum*), potato (*Solanum tuberosum*) and *Arabidopsis thaliana*, to look for *cis*-acting elements responsive to phytohormones in their promoter regions. With the Lsi1 and Lsi2 protein sequences from various plant species, we performed a phylogenetic analysis. Taking into consideration the *Lsi* genes retrieved from tomato, potato and *Arabidopsis*, an expression profiling analysis in different plant tissues was carried out. Expression of Si-regulated genes was also analyzed in response to phytohormones and different plant tissues and developmental stages in *Arabidopsis*.

**Results:**

Si concentrations in plant tissues exhibited the following gradient: roots > stems > leaves. We were able to identify 16 *Lsi1* and three *Lsi2* genes in silico in the pepper genome, while putative *Lsi* homologs were also found in other plant species. They were mainly expressed in root tissues in the genomes analyzed. Both *Lsi* and Si-regulated genes displayed *cis*-acting elements responsive to diverse phytohormones. In *Arabidopsis*, Si-regulated genes were transcriptionally active in most tissues analyzed, though at different expressed levels. From the set of Si-responsive genes, the *NOCS2* gene was highly expressed in germinated seeds, whereas *RABH1B*, and *RBCS-1A*, were moderately expressed in developed flowers. All genes analyzed showed responsiveness to phytohormones and phytohormone precursors.

**Conclusion:**

Pepper root cells are capable of absorbing Si, but small amounts of this element are transported to the upper parts of the plant. We could identify putative Si influx (Lsi1) and efflux (Lsi2) channels that potentially participate in the absorption and transport of Si, since they are mainly expressed in roots. Both *Lsi* and Si-regulated genes exhibit *cis*-regulatory elements in their promoter regions, which are involved in phytohormone responses, pointing to a potential connection among Si, phytohormones, plant growth, and other vital physiological processes triggered by Si in pepper.

## Introduction

Silicon (Si), the 14th element in the periodic table, is the seventh most common element in the universe and the second most abundant element on Earth (making up 28–32% of its mass), while silicon dioxide (SiO_2_) is the most abundant compound in the Earth’s crust (Krebs, 2006). However, plants cannot utilize Si in the form of SiO_2_ (Zargar et al., 2019), with silicic acid or mono silicic acid (Si(OH)_4_ or H_4_SiO_4_) being the Si forms that plants can take up (Mitani, Jian & Iwashita, 2005; Sahebi et al., 2015; Zargar et al., 2019). After absorbed, Si is transported and deposited mainly in the cell apoplast, since Si transport and distribution follows that of water (Handreck & Jones, 1968), thus potentially influencing the physical and chemical properties of the apoplast (Rogalla & Römheld, 2002; Coskun et al., 2019a).

Silicon concentrations in plant tissues exhibit a large variability within and among plant species, which may fluctuate from 0.1% to 10% of the plant biomass on a dry matter basis, depending primarily on the species (Epstein, 1994; Epstein, 1999; Hodson et al., 2005) and secondly on soil properties as a source of Si (Coskun et al., 2019a). Among angiosperms, monocots are the group of plants displaying the highest Si concentrations and dicots are generally less effective at absorbing and transporting Si within their cells and tissues (Guntzer, Keller & Meunier, 2012).

It is worth mentioning that seven out of the ten most produced crops in the world (ranked by quantity) are Si accumulators (Guntzer, Keller & Meunier, 2012) and most of them positively respond to Si applications (Gómez-Merino & Trejo-Téllez, 2018). These crops include rice (*Oryza sativa*; with 4.18% Si on a dry matter basis), wheat (*Triticum aestivum*; 2.45% Si), barley (*Hordeum vulgare*; 1.82% Si), sugarcane (*Saccharum* spp.; 1.60% Si), soybean (*Glycine max*; 1.40% Si), and sugarbeet (*Beta vulgaris* subsp. *vulgari*s; 1.26% Si) (Elsokkary, 2018; Guntzer, Keller & Meunier, 2012; Artyszak, Gozdowski & Kucińska, 2019). Nevertheless, the mechanisms underlying the acquisition and use of Si within plant cells are not yet well understood. Consequently, the role of Si in physiological, biochemical and molecular processes of plants deserves further attention.

Silicon acquisition and transport within the plant cells are carried out by various influx (Lsi1) and efflux (Lsi2) channels, which are encoded by specific genes active in different cell compartments (Elsokkary, 2018; Kaur & Greger, 2019). The presence and activity of Lsi1 and Lsi2 channels determine the capacity of plants to uptake and transport Si (Ma & Yamaji, 2015; Manivannan & Ahn, 2017). In tomato both types of Si channels have been identified (Ma & Yamaji, 2015). However, tomato exhibits no tendency towards saturation kinetics in total Si uptake, suggesting a lack of the active component of Si uptake in the cortex of this species (Nikolic et al., 2007). Since only a very small part (less than 10%) of total root Si can be found in the symplast, tomato is considered a Si excluder species (Heine, Tikum & Horst, 2005). Indeed, a recent study demonstrated that the tomato influx channel SlLsi1 was constitutively expressed and enzymatically active in the roots, whereas the two SlLsi2-like proteins found did not show efflux transport activity for Si (Sun et al., 2020). In pepper, Si has been proved to enhance shoot length and diameter, root length, number of roots, fresh and dry biomass weight, net photosynthesis, stomatal conductance, transpiration rate, antioxidant activity and stomatal opening, stimulating the accumulation of 40 proteins involved in several metabolic processes (Manivannan et al., 2016). Coincidentally, we also found improved growth and metabolism in pepper plants treated with Si (Trejo-Téllez et al., 2020). Nonetheless, Lsi1 and Lsi2 homologs have not been reported in pepper so far. Whether those influx and efflux channels are widespread in other crop plants (including chilies and peppers) and how these channels interact with other genes and proteins remain open questions.

In diverse horticultural crops, the positive effects of Si as a biostimulant have been described (Savvas & Ntatsi, 2015), although the mechanisms through which this element can stimulate metabolic changes in plants are not fully understood (Luyckx et al., 2017a; Muneer et al., 2017; Pontigo et al., 2017). In particular, the effect of Si on phytohormone biosynthesis and degradation has been poorly explored (Luyckx et al., 2017a), though there is evidence that Si may increase cytokinin biosynthesis thus delaying senescence (Markovich et al., 2017). As well, a synergic interaction between Si and gibberellic acid has been found (Khan et al., 2020). In the present work, we grow pepper plants in the presence of different levels of Si in order to analyse the concentrations of this element in roots, stems and leaves. Moreover, we explored the pepper genome in order to identify putative Si channels (Lsi) potentially involved in Si uptake and transport. The corresponding promoter regions of the putative *Lsi* genes were also identified and characterized in silico, while all relevant *cis*-acting elements and putative motifs responsive to phytohormones were determined to prove the role of these molecules on the growth stimulation triggered by Si. As well, we looked for Lsi homologs in other plant species and performed a phylogenetic analysis. Moreover, taking into consideration 40 proteins differentially expressed in response to Si in pepper previously reported by Manivannan et al. (2016), we performed a more in-depth analysis of the promoter regions of the corresponding genes. Since we were not able to find any platforms or databases containing information on pepper gene expression profiles, we took advantage of the eFP Browser tool from the Bio-Analytic Resource (http://bar.utoronto.ca/) (Winter et al., 2007; Waese & Provart, 2016; Waese et al., 2017) to explore expression data of homolog *Lsi* genes from tomato (http://bar.utoronto.ca/efp_tomato/cgi-bin/efpWeb.cgi) and potato (http://bar.utoronto.ca/efp_potato/cgi-bin/efpWeb.cgi), two phylogenetically closely related species to pepper, all of them belonging to the family Solanaceae. We also analyzed data on expression profiles of *Lsi* and Si-regulated homolog genes in *Arabidopsis thaliana* in different tissues, developmental stages and phytohormones through the eFP Brower (http://bar.utoronto.ca/efp/cgi-bin/efpWeb.cgi) and the Genevestigator platform (https://genevestigator.com/gv/index.jsp) (Hruz et al., 2008; Zimmermann et al., 2014). With this, we aimed at seeking *cis*-acting elements and motifs responsive to phytohormones, in order to elucidate a possible connection between the enhanced growth stimulated by Si and plant growth regulators, which may potentially affect other vital physiological processes such as water relations, photosynthetic gas exchange, carboxylation, and nutrient balance, among others.

## Materials and Methods

### Plant material, growing conditions and experimental data analyses

The study was carried out in a greenhouse under the experimental conditions previously described (Trejo-Téllez et al., 2020) and summarized herein for the reader’s convenience. Briefly, thirty-d-old pepper (*Capsicum annuum*) cv. Mysterio seedlings were transplanted into a hydroponic system using the Steiner nutrient solution (Steiner, 1984) at 20% of the original strength (pH 5.5), which was supplemented with micronutrients. Plants were maintained in those conditions for seven days as an adaptation period. After that, the nutrient solution was completely replaced and treatments were applied in the renewed nutrient solution. During the whole experiment, the nutrient solution was aerated every 2 h for 15 min, and totally replaced every seven days. The Si treatments consisted of 60, 125 and 250 mg L^−1^ Si (calcium silicate, CaSiO₃), while the control treatment was the Steiner nutrient solution without adding Si.

A completely randomized experimental design was used, where a single plant, with 12 replicates per treatment, represented the experimental unit. Once normality and homogeneity of the variances were proven, a one-way analysis of variance (ANOVA) was performed. Means were compared using the Duncan test with α = 0.05. All analyses were conducted using the SAS software (SAS, 2011).

### Measurements of Si concentrations in leaves, stems and roots of pepper plants

The concentrations of silicon (Si) were determined in the dry biomass of leaves, stems, and roots. A wet acid digestion was used with a mixture of perchloric and nitric acids (Alcántar & Sandoval, 1999). Subsequently, samples were analyzed with an inductively coupled plasma optical emission spectrometer (Agilent 725 ICP-OES; Mulgrave, VI, Australia).

### Identification of Si channels and Si-regulated genes in pepper

In order to identify putative Si channels in pepper, we used the protein sequences of the silicon channels Lsi1 (Ma et al., 2006) and Lsi2 (Ma et al., 2007) identified in rice, as well as Lsi1 and Lsi2 homologs reported in strawberry (*Fragaria* x *ananassa*) (Ouellette et al., 2017). The multiple alignments for the comparison of protein sequences found were done using the Protein BLAST program from NCBI (https://blast.ncbi.nlm.nih.gov/Blast.cgi) (Altschul et al., 1990) and UniProtKB (The UniProt Consortium, 2019), with all the default parameters already set. Moreover, taking into consideration the set of Si-regulated genes previously reported by Manivannan et al. (2016), we further searched for those genes and analyzed their promoter regions and expression profiles in silico in different plant species. We listed the Lsi proteins identified in silico in the pepper genome, and complemented the information on Si-regulated genes. Detailed data and information regarding these analyses are enclosed as File SF1.

### Phylogenesis and domain analyses of Lsi channels

With the protein sequences of pepper (*Capsicum annuum*) Lsi channels, henceforth denoted as CaLsi1 and CaLsi2, and their homologs identified in other plant species, we performed a phylogenetic analysis. To this end, first we performed a multiple sequence alignment by using the Clustal Omega software from EMBL-EBI (ebi.ac.uk/Tools/msa/clustalo) (Sievers et al., 2011). Then, the identification and corresponding mapping of the conserved domains found in the CaLsi1 and CaLsi2 proteins were carried out by using the HMMER3.2.1 software (Potter et al., 2018).

### Acquisition of promoter sequences

The lists of *Lsi* and Si-regulated genes were necessary and sufficient to obtain their respective promoters. These nucleotide sequences were downloaded from NCBI through the Gene database (www.ncbi.nlm.nih.gov/gene/), considering 2,000 base pairs (bp) upstream of the coding region. Promoter sequences of the *Lsi* genes are listed in File SF2, while those of the Si-regulated genes are included in File SF3. In addition, the promoter sequences of genes encompassing an external group are included in File SF4. This external group of genes was considered in our promoter analysis in order to validate our data for each species analyzed, since they encode proteins other than Lsi channels, and are mainly expressed in the shoot system (with lower or no transcriptional activity in the root system as compared to the *Lsi* genes).

### Promoter analysis

Taking into consideration the nucleotide sequences of Si channels, Si-regulated genes, and the set of genes considered as an external group, the corresponding *cis*-acting elements in each promoter were revealed through the PlantCARE database (http://bioinformatics.psb.ugent.be/webtools/plantcare/html/) (Lescot et al., 2002). According to the differential gene expression reported by Manivannan et al. (2016), the sequences were divided into induced and repressed genes. Since we quantified the frecuency of the motif sequences, their distribution in the promoter was not considered as a parameter in our analysis.

### Analysis of gene expression

Expression profiles of *Lsi* homolog genes in tomato, potato and *Arabidopsis* in different tissues were retrieved from the eFP Browser tool from the Bio-Analytic Resource (http://bar.utoronto.ca/affydb/BAR_instructions.html) (Waese & Provart, 2016; Waese et al., 2017). Furthermore, for *Arabidopsis*, expression data on *Lsi* homolog genes according to developmental stages and environmental stimuli under the “Hormone” section were analyzed through the Genevestigator platform (https://genevestigator.com/) (Hruz et al., 2008; Zimmermann et al., 2014). It is worth mentioning that for this purpose, only data from *Arabidopisis thaliana* (as a eudicot) were taken into consideration, since no information on pepper could be retrieved from our search.

## Results

### Silicon concentrations in leaves, stems and roots

**Figure 1:**
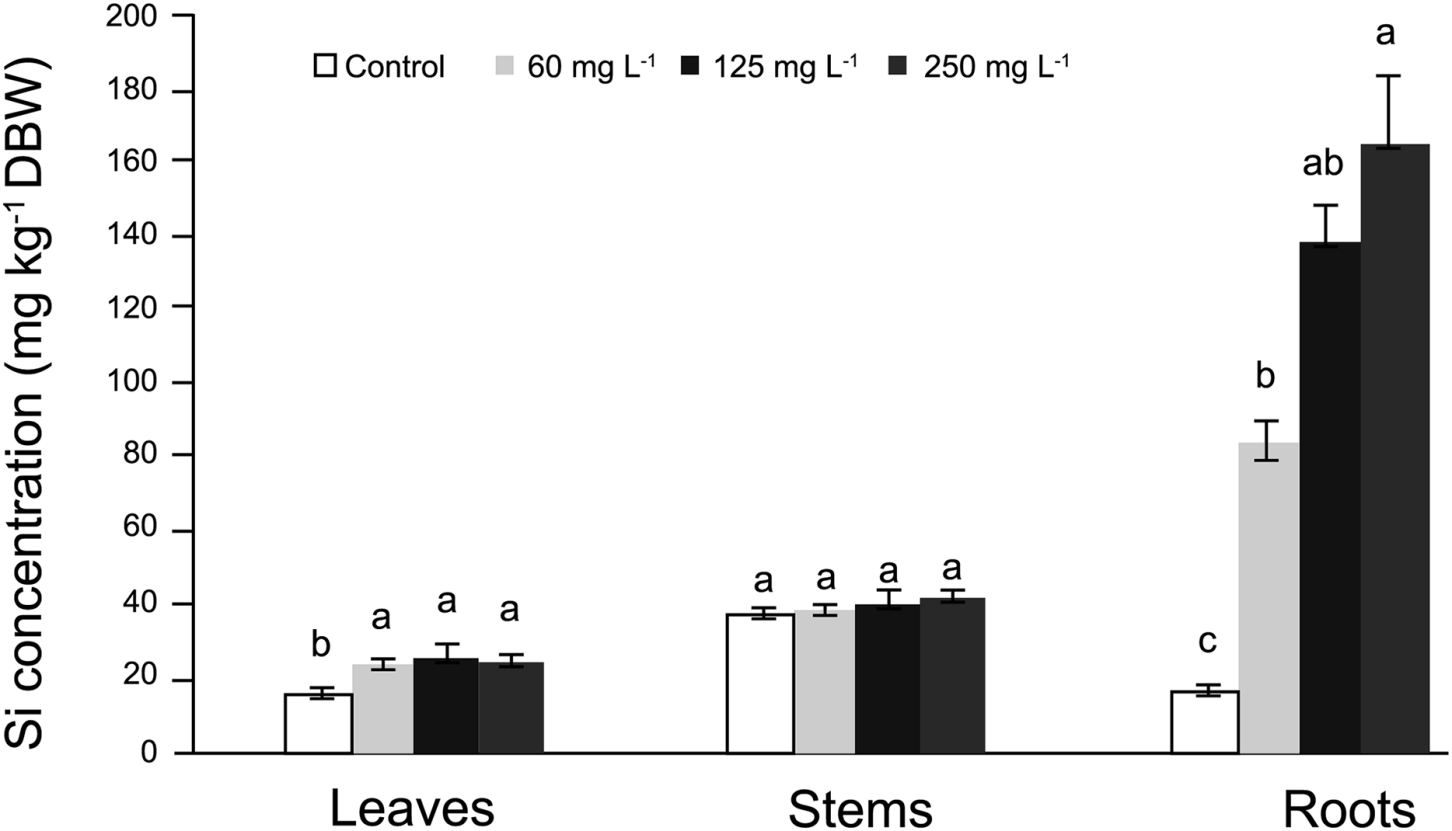
Concentration of silicon (Si) in leaves, stems, and roots of pepper (*Capsicum annuum*) plants grown in a nutrient solution with different concentrations of Si for 28 days. Error bars indicate standard deviation. Columns with different letters are statistically different (*P* ≤ 0.05).

In roots, the average Si concentrations in Si-treated plants was nearly 130 mg kg^−1^ Si on a dry basis, while stems had nearly 37 mg kg^−1^ Si and leaves approximately 31 mg kg^−1^ Si. In general, in control plants Si concentrations fluctuated from 18 to 35 mg kg^−1^ Si (Fig. 1). In roots, Si concentrations were significantly higher in plants supplied with Si; as the Si concentration increased, the Si levels in the roots also increased. In stems, no significant differences were observed among treatments, while in leaves, higher concentrations were recorded in Si-treated plants as compared to the control. Detailed data on the corresponding analyses are enclosed as SF1 Raw Data (for roots, stems and leaves).

### Identification of putative Si influx and efflux channels

We have previously demonstrated that Si induces hormetic dose-response effects on growth and metabolism in pepper plants during the early developmental stage (Trejo-Téllez et al., 2020), which coincides with other findings previously reported for this species (Manivannan et al., 2016). Hence, in this study we aimed to determine if this species was indeed a Si-competent one following the recent characterization of the properties of Si channels. Based on genomic data, we were able to conclude that pepper contains both influx (Lsi1) and efflux (Lsi2) channels for Si uptake and transport.

The protein sequences were obtained by using the Protein BLAST program from NCBI (blast.ncbi.nlm.nih.gov/Blast.cgi) and UniProtKB (The UniProt Consortium, 2019) taking into consideration Lsi proteins exhibiting the highest homology to the strawberry FaNIP2-1 and FaLsi2 genes previously reported (Ouellette et al., 2017). As a result, the amino acid sequences of 16 Lsi1 influx channels were obtained: CaLsi1.1, CaLsi1.2, CaLsi1.3, CaLsi1.4, CaLsi1.5, CaLsi1.6, CaLsi1.7, CaLsi1.8, CaLsi1.9, CaLsi1.10, CaLsi1.11, CaLsi1.12, CaLsi1.13, CaLsi1.14, CaLsi1.15 and CaLsi1.16. As well, we were able to retrieve three Lsi2 efflux channels from these platforms: CaLsi2.1a, CaLsi2.1b and CaLsi2.3 (Table 1). From the sequences of each one of the aforementioned proteins, the corresponding Lsi homologs were obtained for tomato, potato, *Arabidopsis*, maize (*Zea mays*), rice and sorghum (*Sorghum bicolor*) (Table 1). Selection criterion for each homolog protein was defined taking into consideration two variables: (1) the Percentage of Query Coverage (%QC) and the Percentage of Maximum Identity (%ID). For instance, in the case of CaLsi1 we selected the %QC and %ID equal or higher than 90% (%QC >= 90; %ID >= 90) for tomato and potato; in *Arabidopsis*, %QC = 100 and %ID >= 70; in maize, %QC >= 98 and %ID >= 79.8; in rice, %QC = 100 and %ID >= 70; in sorghum, %QC >= 80 and %ID >= 70. In the case of CaLsi2, we obtained %QC >= 83 and %ID >= 75 for potato; in tomato, %QC >= 83 and %ID >= 74; in *Arabidopsis*, %QC >= 99 and %ID >= 68; in maize, rice and sorghum, %QC >= 99 and %ID >= 60. In our phylogenetic analysis, apart from the 16 CaLsi1 and three CaLsi2 proteins, we included 20 homolog sequences for CaLsi1 and 30 homolog sequences for CaLsi2, which were retrieved from the tomato, potato, *Arabidopsis*, maize, rice and sorghum proteomes. File SF1 comprises the full list of Lsi channels we found in our search.

**Table 1:** List of putative Low silicon influx (Lsi1) and efflux (Lsi2) channels identified in silico in the pepper genome and their homologs in potato, tomato, *Arabidopsis*, maize, rice and sorghum.

Gene name*	Gene locus	GenBank/UniProtKB accession	Protein length (aa)	Molecular mass (kDa)	cDNA/EST/mRNA
*CaLsi1.1*	XP_016559617.1	XM_016704131.1/A0A2G3A7U2	274	28.95	LOC107859207
*CaLsi1.2*	XP_016570571.1	XM_016715085.1/A0A1U8GGZ8	284	30.17	LOC107868397
*CaLsi1.3*	PHT61911	PHT61911.1/A0A2G2XX71	283	30.11	T459_34224
*CaLsi1.4*	XP_016556948.1	XM_016701462.1/A0A1U8FKV2	251	26.92	LOC107856450
*CaLsi1.5*	XP_016556947.1	XM_016701461.1/A0A1U8FC86	271	29.06	LOC107856450
*CaLsi1.6*	PHT88450.1	PHT88450.1/A0A2G3A2M0	305	31.37	T459_10556
*CaLsi1.7*	XP_016562905.1	XM_016707419.1/A0A1U8FVN4	305	31.43	LOC107861995
*CaLsi1.8*	PHT79955.1	PHT79955.1/A0A2G2ZDK0	290	31.63	T459_18007
*CaLsi1.9*	XP_016561864.1	XM_016706378.1/A0A1U8G1K6	280	29.76	LOC107860944
*CaLsi1.10*	XP_016556163.1	XM_016700677/A0A2G3APC1	297	30.92	LOC107855657
*CaLsi1.11*	XP_016576910.1	XM_016721424.1/A0A1U8H0K7	344	37.35	LOC107874665
*CaLsi1.12*	XP_016560837.1	XM_016705351.1/A0A2G3AAT2	264	28.07	LOC107860109
*CaLsi1.13*	PHT88955.1	PHT88955.1/A0A2G3A3Z3	295	31.48	T459_04068
*CaLsi1.14*	PHT67190.1	PHT67190.1/A0A2G2YBT3	191	20.57	T459_26677
*CaLsi1.15*	PHT90298.1	PHT90298.1/A0A2G3A7W5	246	26.44	T459_05411
*CaLsi1.16*	XP_016558304.1	XM_016702818.1/A0A1U8FRD1	307	33.07	LOC107858088
*StNIP1.2*	XP_006356091.1	XM_006356029.2/M1CNX1	277	29.65	LOC102581997
*StNIP1.3*	XP_006344325.1	XM_006344263.1/M1CZX0	272	29.26	LOC102585910
*StNIP6.1*	XP_006341363.1	XM_006341301.2/M1B3X8	306	31.68	LOC102599870
*StNIP5.1*	NP_001274996.1	NM_001288067.1/H9BZ83	296	30.67	NM_001288067.1
*SlNIP1.1*	XP_004234073.1	XM_004234025.4/A0A3Q7FD37	277	29.65	LOC101246598
*SlNIP1x2*	XP_010320707.2	XM_010322405.3	272	29.28	LOC101252874
*SlNIP6.1*	NP_001289852.1	NM_001302923.1/V5YMM2	306	31.71	LOC101243873
*SlNIP5.1*	NP_001274288.1	NM_001287359.2/K4CJ08	295	30.63	LOC101244210
*AtUPP*	CAA0344932.1	CACSHJ010000087.1/A0A5S9WW53	305	31.91	C24_LOCUS7011
*AtNOD26.6*	NP_178191.1	NM_106724.3/Q9SAI4	305	31.84	AT1G80760
*AtNOD26.5*	NP_192776.1	NM_117106.3/Q9SV84	304	31.49	AT4G10380
*ZmUPP*	ACN36318.1	BT069421.1/C0PMA2	302	31.14	BT069421.1
*ZmNIP3.1*	NP_001105021.1	NM_001111551.1/Q9ATN1	302	31.16	LOC541885
*OsNIP3.1*	XP_015614995.1	XM_015759509.2/Q0IWF3	311	31.84	LOC4349102
*OsHYP1*	KAB8113302.1	CM012950.1	311	31.89	EE612_052268
*OsHYP2*	EAZ16668.1	CM000147.1/Q0IWF3	309	31.84	OsJ_32143
*OsNOD26.3*	BAO01152.1	AB856414.1/U3U951	309	31.58	AB856414.1
*SbNIP1.1*	XP_002453573.1	XM_002453528.2/C5XYL0	287	30.19	LOC8073813
*SbNIP1.2*	XP_002454982.2	XM_002454937.2/A0A1B6Q107	282	29.26	LOC8080506
*SbNIP1.3*	XP_002440774.1	XM_002440729.2/C5YUM4	283	29.49	LOC8055735
*CaLsi2.1a*	XP_016564559.1	PHT87291.1/XM_016709074.1	532	57.8	LOC107863243
*CaLsi2.1b*	XP_016564559.1	XM_016709073.1/A0A1U8G953	532	57.8	LOC107863243
*CaLsi2.3*	XP_016564560.1	XP_016564560.1	491	52.9	LOC107863243
*StARSBx1*	XP_006363328.1	XM_006363266.2/M1CXN0	533	57.91	LOC102599999
*StARSBx2*	XP_015158879.1	XM_015303393.1	452	49.45	LOC102599999
*SlLsi2*	XP_010317628.1	XM_010319326.3/A0A3Q7FEL4	516	56.3	LOC101259123
*SlLsi2x1*	XP_004240725.1	XM_004240677.4/A0A3Q7GUH1	528	57.33	LOC101263323
*SlLsi2x3*	XP_025887220.1	XM_026031435.1	465	50.98	LOC101263323
*SlLsi2x2*	XP_025887219.1	XM_026031434.1	466	51.11	LOC101263323
*AtUPP1*	CAA0156383.1	CACSHJ010000087.1	502	54.65	C24_LOCUS167
*AtUPP2*	VYS44749.1	CACRSJ010000104.1/A0A654E638	499	54.48	AN1_LOCUS259
*AtDIS*	NP_001320653.1	NM_001331329.1/A0A384KQY8	502	54.72	AT1G02260
*ZmUP1*	NP_001151517.1	NM_001158045.1/B6U1B5	556	60.20	LOC100285151
*ZmCTFP1*	ACG43196.1	EU971078.1/B6U1G3	556	60.21	EU971078.1
*ZmUP2*	NP_001147885.1	NM_001154413.2/B6SSI8	574	61.39	LOC100281495
*ZmARSB1*	PWZ55837.1	NCVQ01000001.1/A0A317YAZ4	574	61.41	Zm00014a_001976
*ZmUP3*	XP_008679680.1	XM_008681458.2/B8A307	535	58.04	XM_008681458
*ZmCTFP2*	ACG28034.1	EU955916.1/B6ST51	575	61.46	EU955916.1
*ZmARSB2*	PWZ22675.1	NCVQ01000006.1/A0A3L6ENZ5	550	59.09	Zm00014a_004231
*ZmLsi2x2*	XP_008644817.1	XM_008646595.3/A0A1D6GPX1	550	59.11	LOC103626193
*ZmLsi2x1*	XP_008644816.1	XM_008646594.2	653	70.77	LOC103626193
*ZmARSB3*	PWZ25127.1	NCVQ01000005.1/A0A3L6F0E9	527	56.19	Zm00014a_028813
*ZmATSB*	NP_001148124.1	NM_001154652.2/B6SY85	527	56.23	LOC100281732
*OsLsi2x1*	XP_015630657.1	XM_015775171.2/Q10RS7	557	60.24	LOC4331617
*OsLsi2x2*	XP_015630663.1	XM_015775177.2/Q10RS6	527	57.11	LOC4331617
*OsLsi2.2*	XP_015614851.1	XM_015759365.2/A0A0P0XUU1	567	61.36	LOC4348760
*OsLsi2.3*	XP_015626922.1	XM_015771436.2/Q6K6Z6	530	56.78	LOC4331184
*OsHYP3*	KAB8089565.1	CM012942.1	530	56.8	EE612_014533
*OsHYP4*	EEC74268.1	CM000127.1/B8AF47	485	51.78	OsI_09493
*SbARSB1x1*	XP_021307248.1	XM_021451573.1	556	60.24	LOC110431862
*SbARSB1*	XP_021306519.1	XM_021450844.1	564	60.84	LOC8071382
*SbARSB1x2*	XP_021307254.1	XM_021451579.1	535	58.07	LOC110431862
*SbARSB2*	XP_002453063.1	XM_002453018.2/C5XW09	494	52.83	LOC8059699

**Note:**

Data were retrieved from NCBI and UniProtKB. *For each *Lsi* gene name, we considered the scientific name of pepper: *Capsicum annuum* (*Ca*); potato: *Solanum tuberosum* (*St*); tomato: *Solanum lycopersicum* (*Sl*); *Arabidopsis*: *Arabidopsis thaliana* (*At*); maize: *Zea mays* (*Zm*); rice: *Oryza sativa* (*Os*); and sorghum: *Sorghum bicolor* (*Sb*). *The meaning of the acronym for Lsi homologous proteins is as follows: Nodulin 26-like Intrinsic Protein (NIP), Unnamed Product Protein (UPP), Nucleotide Oligomerization Domain (NOD), Hypothetical Protein (HYP), Arsenical Pump Membrane Protein (ARSB), Divalent Ion Symporter (DIS), Uncharacterized Protein (UP), Citrate Transporter Family Protein (CTFP), Arsenite Transport Subunit B (ATSB). This table presents the Lsi1 channels with the highest homology to strawberry FaNIP2-1 and FaLsi2 proteins previously reported (Ouellette et al., 2017) and their homologs. The complete list of putative Lsi and Lsi2 channels is included as File SF1.

### Phylogenetic analysis and domain organization of Lsi channels

**Figure 2:**
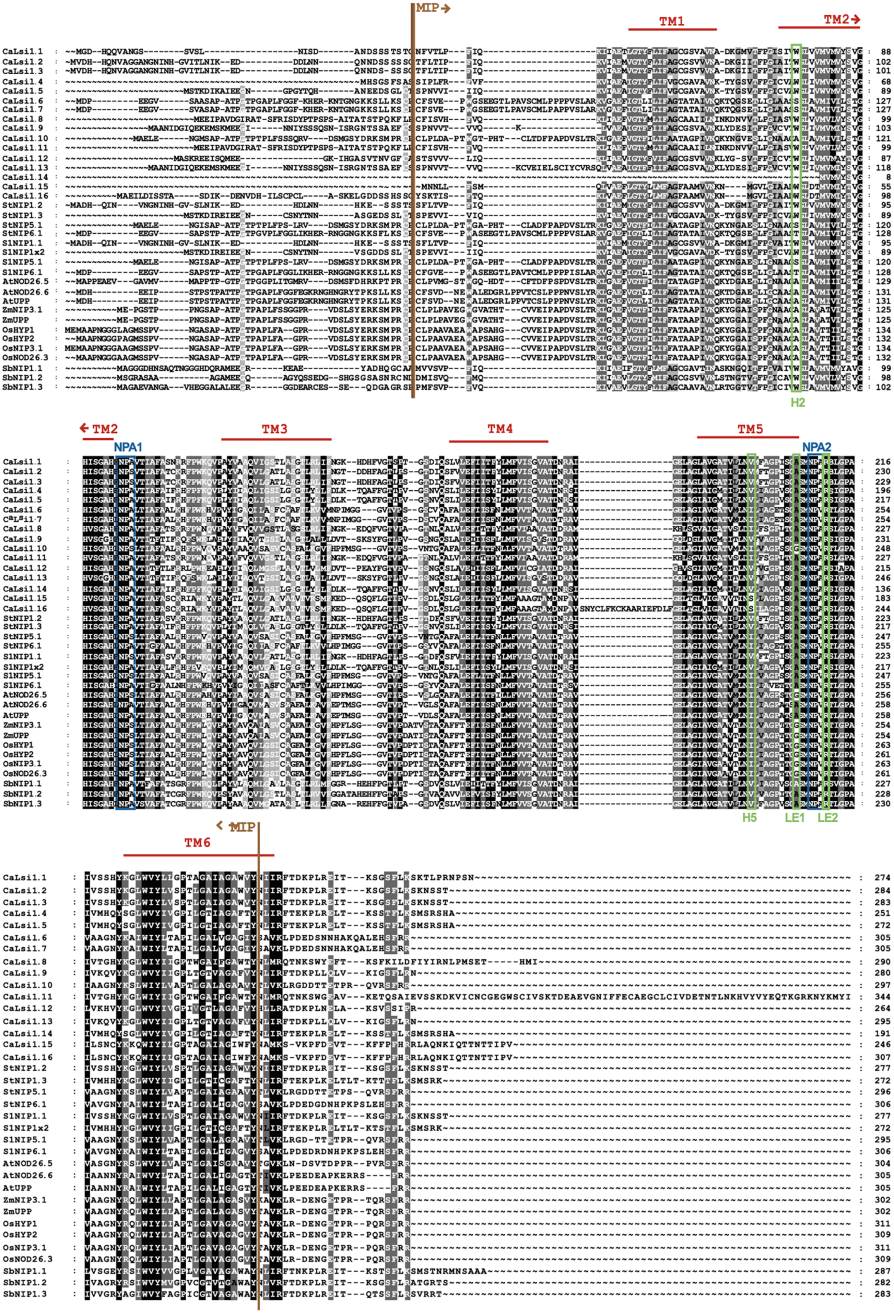
Protein sequence alignment of pepper CaLsi1 channels and their homologs. The Major Intrinsic Protein (MIP) domain and six Transmembrane (TM) domains are indicated. Two Asn-Pro-Ala (NPA) motifs and a conserved aromatic/arginine (ar/R) selective filter, made up at the secondary structure level of the helix 2 (H2), helix 5 (H5), loop E1 (LE1) and loop E2 (LE2), were also detected in most Lsi1 sequences analyzed, but the (G/A)SGR motif, which is crucial to enhance the capacity of plant species to accumulate Si, was not present. The dashes represent gaps introduced to optimize the alignment. Motif/residues discussed in the text are highlighted in different colors.

**Figure 3:**
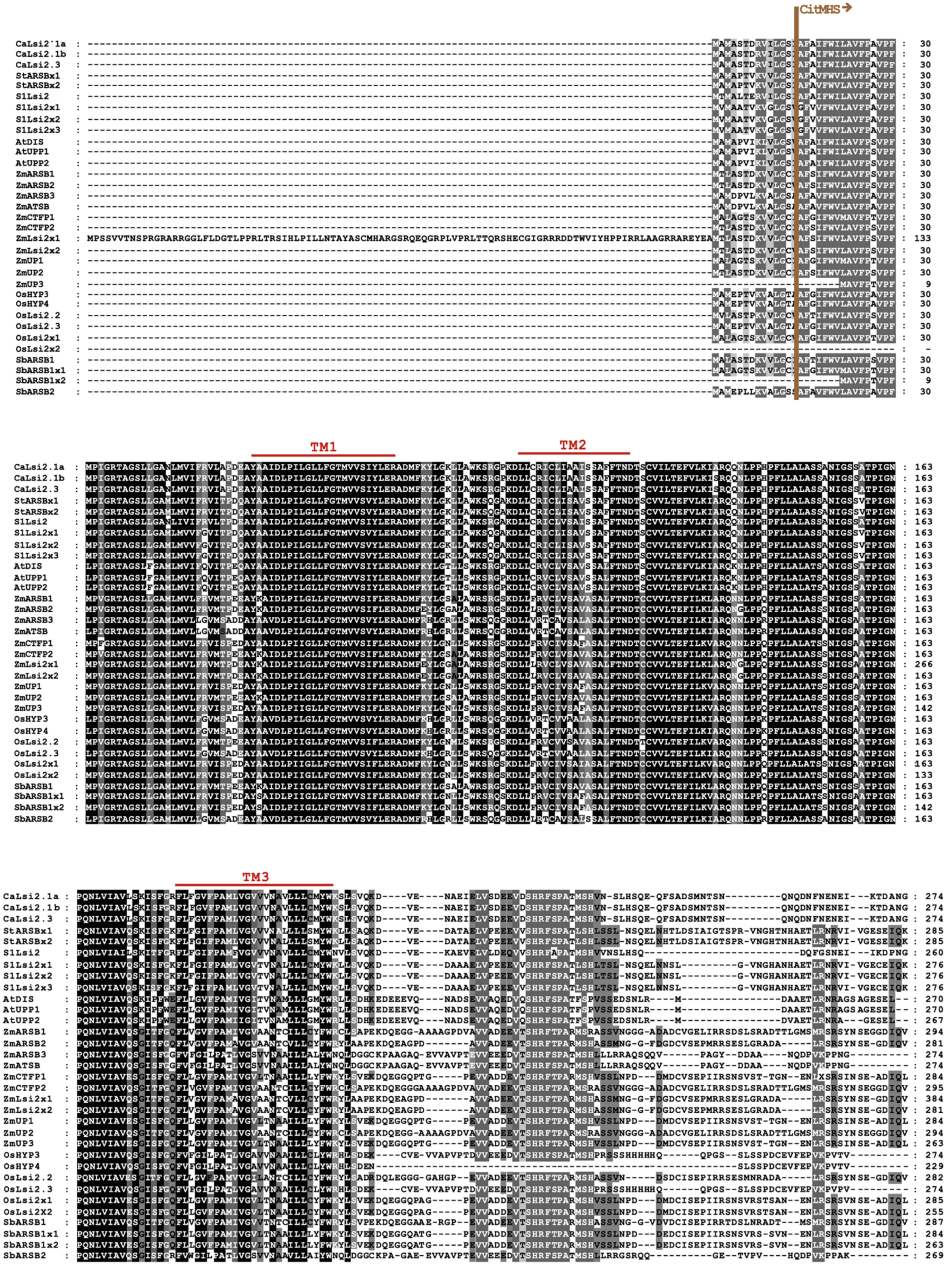
Protein sequence alignment of pepper CaLsi2 channels and their homologs Part I. The start of the CitMHS domain and three (TM1-TM3) of the nine conserved TM domains. The dashes represent gaps introduced to optimize the alignment. Motif/residues discussed in the text are highlighted in different colors.

Once we obtained the protein sequences of CaLsi1, CaLsi2 and their homologs, we performed a phylogenetic analysis with all sequences listed in Table 1. To this end, we first performed a multiple sequence alignment by using the Clustal Omega software from EMBL-EBI (ebi.ac.uk/Tools/msa/clustalo) (Sievers et al., 2011). The corresponding results from this analysis are shown in Fig. 2. The identification and corresponding mapping of the conserved domains found in the CaLsi1 and CaLsi2 proteins were carried out by using the HMMER3.2.1 software (Potter et al., 2018). All CaLsi1 sequences and their homologs contain a Major Intrinsic Protein (MIP) domain and six Transmembrane (TM) domains, with the exception of CaLsi1.14 that contains only five TM domains. Furthermore, in CaLsi1, we could detect the presence of a conserved aromatic/arginine (ar/R) selective filter and two Asn-Pro-Ala (NPA) motifs, with the exception CaLsi1.14 that only contains a single NPA (Fig. 3). These two motifs play a critical role in the selectivity of Si by the Lsi channels (Deshmukh et al., 2015; Vatansever et al., 2017). Nevertheless, we could observe that not one of the CaLsi1 sequences analyzed contained the (G/A)SGR motif, which is present in the Lsi1 sequences of high and moderate Si accumulators. Therefore, the species we analyze herein can be categorized as low Si accumulators. After the protein sequences were aligned and a detailed phylogenetic analysis was carried out, we could detect two major clusters based on the amino acid residues that form part of the ar/R filter (Fig. 5). Cluster I encompasses the protein sequences containing the WVAR motif: CaLsi1.4, CaLsi1.5, StNIP1.3, SlNIP1x2, CaLsi1.12, CaLsi1.9, CaLsi1.13, SbNIP1.2, SbNIP1.3, SbNIP1.1, CaLsi1.1, CaLsi1.2, CaLsi1.3, StNIP1.2 and SlNIP1.1; the exception in this cluster was CaLsi1.14, which lacks the first amino acid residue W. Cluster II comprises the following motifs: WIAR (CaLsi1.8 and CaLsi1.11), WSAR (CaLsi1.15 and CaLsi1.16), AIAR (AtUPP and AtNOD26.6), SIAR (CaLsi1.6, CaLsi1.7, StNIP5.1 and SlNIP5.1), TIAR (StNIP6.1 and SlNIP6.1) and AIGR (AtNOD26.5, CaLsi1.10, ZmUPP, ZmNIP3.1, OsHYP1, OsNIP3.1, OsNOD26.3 and OsHYP2) (Figs. 3 and 5).

**Figure 4:**
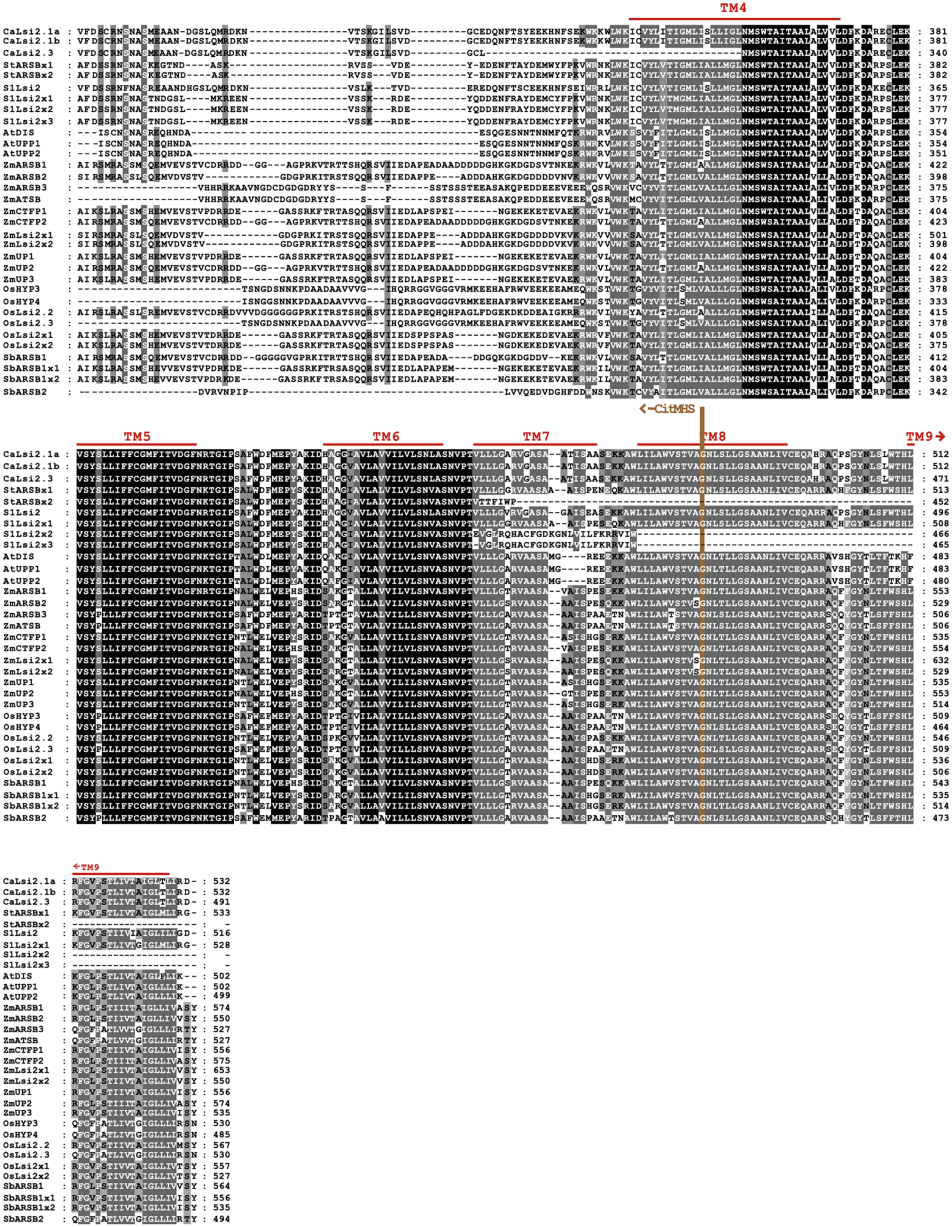
Protein sequence alignment of pepper CaLsi2 channels and their homologs Part II. Five (TM4-TM9) of the nine conserved TM domains as well as the end of the CitMHS domain. The dashes represent gaps introduced to optimize the alignment. Motif/residues discussed in the text are highlighted in different colors.

The CaLsi2 proteins exhibit a simpler domain organization as compared to CaLsi1. Indeed, all the CaLsi2 proteins and their homologs contain a Citrate-Mg^2+^:H^+^ (CitM) − Citrate-Ca^2+^:H^+^ (CitH) Symporter (CitMHS) domain (involved in ion transport), and nine conserved TM domains (Fig. 4). The corresponding phylogenetic tree of the CaLsi2 proteins and their homologs is depicted in Fig. 6.

**Figure 5:**
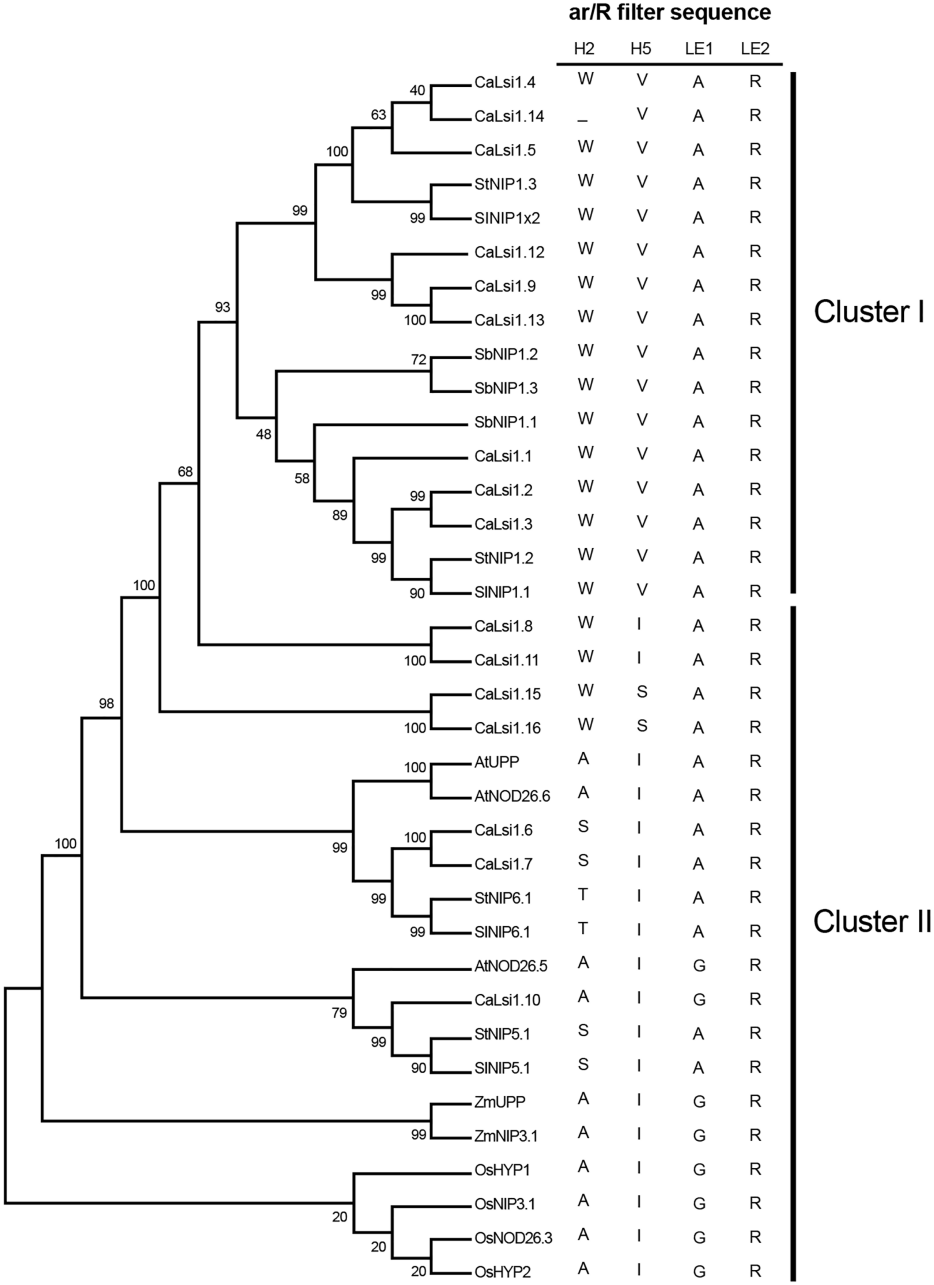
Phylogenetic analysis of the pepper (*Capsicum annuum*) CaLsi1 channels and their homologs. Cluster I encompasses the Lsi1 channels harboring the WVAR conserved domain, while Cluster II comprises the WIAR, WSAR, AIAR, SIAR, TIAR and AIGR motifs. The phylogenetic tree was constructed by using the Neighbor–Joining method (Saitou & Nei, 1987). We used 10,000 bootstrap replicates to assess the robustness of phylogenetic inferences and to place confidence intervals to the model developed through the values indicated in each node (Felsenstein, 1985). The validation analysis was carried out with the Mega7.0 program (Kumar, Stecher & Tamura, 2016).

**Figure 6:**
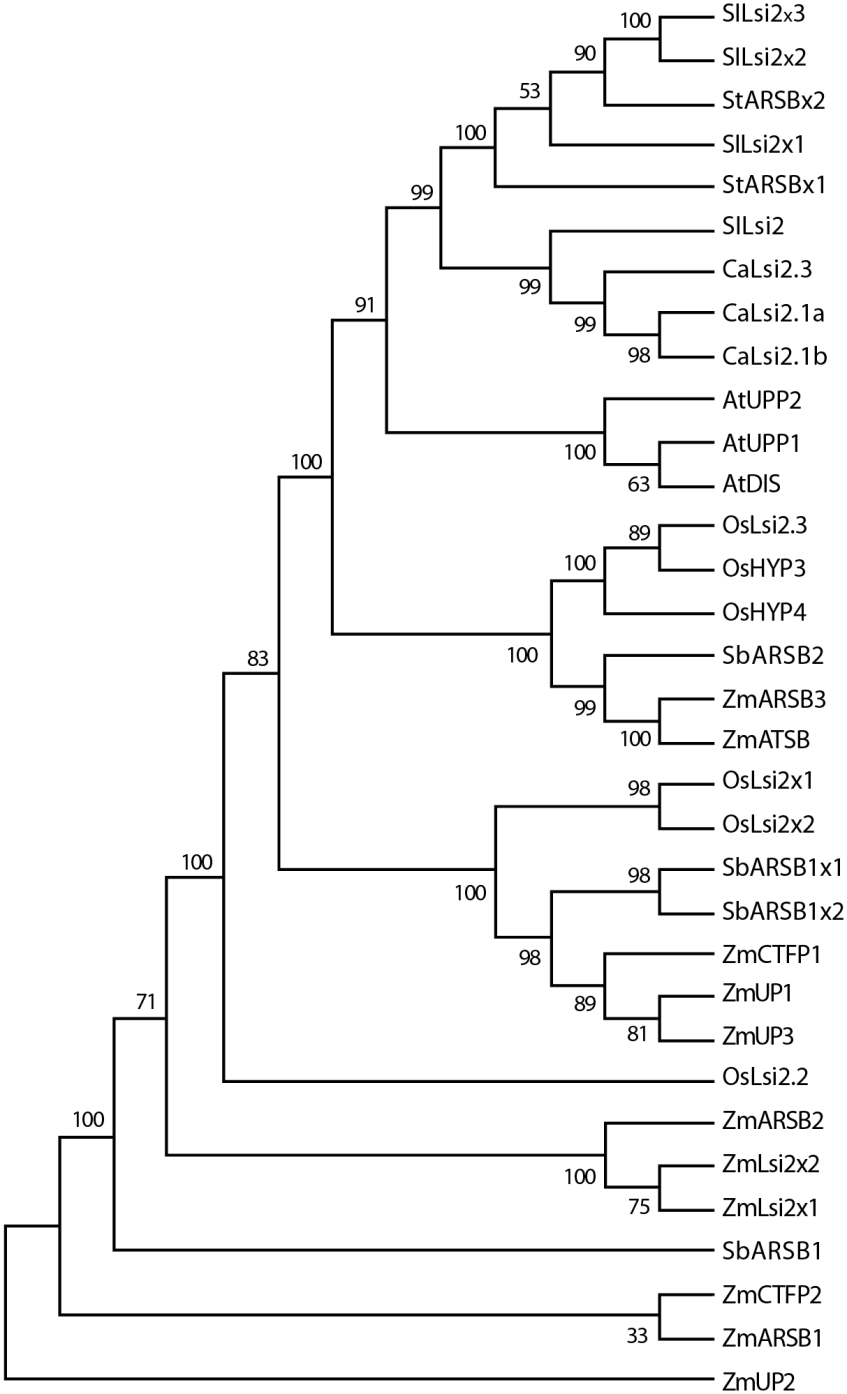
Phylogenetic tree of the pepper (*Capsicum annuum*) CaLsi2 channels and their homologs. The CitMHS domain and nine conserved TM domains are clearly depicted. The phylogenetic tree was constructed by using the Neighbor–Joining method (Saitou & Nei, 1987). We used 10,000 bootstrap replicates to assess the robustness of phylogenetic inferences and to place confidence intervals to the model developed through the values indicated in each node (Felsenstein, 1985). The validation analysis was carried out with the Mega7.0 program (Kumar, Stecher & Tamura, 2016).

### Promoter analysis of the *CaLsi1* and *CaLsi2* genes

Promoter analysis of the *Lsi1* and *Lsi2* genes revealed the presence of *cis*-acting elements involved in response to plant hormones including abscisic acid (ABRE), jasmonic acid (G-box, CGTCA-motif and TGACG-motif), ethylene (ERE), salicylic acid (TCA-element), auxins (AuxRR-core and TGA-element) and gibberellic acid (GARE-motif, P-Box and TATC-Box) (Table 2). The sequences of promoters analyzed in this section are enclosed as File SF2, while File SF5 encompasses additional information on this analysis.

**Table 2:** List of *cis*-regulatory elements responsive to phytohormones found in the promoter regions of *CaLsi1* and *CaLsi2* genes in the pepper (*Capsicum annuum*) genome.

Gene locus	ABRE	Aux-RR-core	CGTCA-motif	ERE	G-box	GARE-motif	P-box	TATC-box	TCA-element	TGA-element	TGACG-motif
XP_016559617	2	–	–	6	2	–	–	–	–	–	–
XP_016570571	4	–	6	–	5	–	–	–	2	1	6
XP_016556948	1	1	–	2	1	–	–	–	–	–	–
XP_016556947	1	1	–	2	1	–	–	–	–	–	–
XP_016562905	3	–	4	3	1	1	1	–	–	–	4
XP_016561864	4	–	2	3	1	–	–	–	1	–	2
XP_016556163	–	–	–	10		–	–	–	–	–	–
XP_016576910	1	–	1	–	1	1	3	–	–	–	1
XP_016560837	6	–	2	–	3	–	–	–	–	1	2
XP_016558304	4	–	1	1	3	–	3	–	–	3	1
XP_016564559	–	–	1	–	–	1	1	5	–	3	1
XP_016564560	–	–	1	–	–	1	1	5	–	3	1

**Note:**

Abscisic acid: ABRE; jasmonic acid: G-box, CGTCA-motif and TGACG-motif; ethylene: ERE; salicylic acid: TCA-element; auxins: AuxRR-core and TGA-element; and gibberellic acid: GARE-motif, P-Box and TATC-Box.

### Promoter analysis of Si-regulated genes

A total of 40 proteins were previously found to be differentially expressed in response to Si in pepper (Manivannan et al., 2016). In general, the gene ontology analysis revealed that these proteins are involved in vital physiological processes, including photosynthesis, carbohydrate metabolism, catabolic processes, protein modification, DNA metabolism, signal transduction, and transport, to name some. A summary of the characteristics of these proteins is included as File SF6. The analysis of the promoter regions of the corresponding genes revealed that some of them exhibit *cis*-acting elements and motifs responsive to phytohormones, which suggests a possible connection between the enhanced growth stimulated by Si and phytohormones. In a similar manner to the elements found in the promoter regions of *Lsi* genes, the corresponding Si-regulated genes exhibited responsiveness to abscisic acid, jasmonic acid, ethylene, salicylic acid, auxins and gibberellic acid (Table 3). File SF2 includes the promoters sequences of Si-regulated genes considered for this analysis, while File SF7 encompasses additional information on this section.

**Table 3:** List of *cis*-regulatory elements responsive to phytohormones found in the promoter regions of some Si-regulate genes in pepper (*Capsicum annuum*).

Gene locus	ABRE	Aux-RR-core	CGTCA-motif	ERE	G-box	GARE-motif	P-box	TATC-box	TCA-element	TGA-element	TGACG-motif
XP_004249273 (*PURA*)	4	–	1	5	2	–	1	–	1	–	1
XP_008793948	4	–	–	–	2	1	1	–	1	–	3
XP_010243911	1	–	1	–	1	–	–	–	–	–	1
XP_010655136	–	–	–	5	–	–	–	–	–	–	–
XP_009393059 (*GME*)	9	–	2	3	6	–	2	–	2	–	2
XP_003058724	9	–	5	–	5	–	–	–	–	1	5
XP_006647478 (*CML5*)	3	–	2	1	2	–	–	–	1	–	2
XP_009398204	4	–	–	–	3	–	–	–	–	–	1
XP_010517956 (*MOCS2*)	5	–	–	–	2	–	3	–	–	1	–
XP_008677250	2	2	2	1	1	–	–	1	3	1	2
XP_010046336	1	1	4	–	–	1	–	–	1	–	4
XP_004951624	4	–	–	1	5	2	1	–	2	–	–
XP_009588622	3	–	–	1	3	1	1	–	1	–	–
XP_004979712	3	–	1	–	1	–	–	–	–	–	1
XP_009147561	2	–	–	5	1	–	–	–	–	–	–
XP_012064817 (*DRB2*)	2	–	–	5	1	–	–	–	–	1	–
XP_006450493	1	–	1	1	1	–	–	1	–	–	1
XP_009588623	3	–	–	1	3	1	1	–	1	–	–
XP_006485364 (*RABH1B*)	1	–	1	–	1	–	–	–	2	–	1
XP_003598507 (*AT3G23880*)	4	–	1	1	3	–	1	–	1	1	1

**Note:**

Abscisic acid: ABRE; jasmonic acid: G-box, CGTCA-motif and TGACG-motif; ethylene: ERE; salicylic acid: TCA-element; auxins: AuxRR-core and TGA-element; and gibberellic acid: GARE-motif, P-Box and TATC-Box. Gene locus were retrieved from Manivannan et al. (2016).

### Promoter analysis of the set of genes comprising the external group

The promoter regions of the set of genes included as an external group for the purpose of this study revealed the presence of *cis*-acting elements responsive to phytohormones. Nonetheless, the frequency of the regulatory elements responsive to gibberellins was lower in the external group as compared to the *Lsi* genes. In fact, only a P-box and a TATC-box were found in the promoter region of the external group in our search (Table 4), while the promoter region of the *Lsi* (Table 2) and the Si-regulated genes (Table 3) contained up to three P-box and 5 TATC-box elements. Indeed, the combined application of Si and gibberellic acid stimulates plant growth and metabolic regulation, and boosts tolerance mechanisms against stress, thus representing a novel sustainable approach aimed at overcoming extreme environmental conditions imposed by global climate change (Khan et al., 2020). Since climate change exerts adverse effects on crop production, the identification of solutions that minimize the negative impacts of climate change on crops are of paramount importance to achieve crop productivity and hence food security (Pareek, Dhankher & Foyer, 2020).

**Table 4:** List of *cis*-regulatory elements responsive to phytohormones found in the promoter regions of the external group of genes in *Arabidopsis* (*Arabidopsis thaliana*).

Gene locus	ABRE	Aux-RRcore	CGTCA-motif	ERE	G-box	GARE-motif	P-box	TATC-box	TCA-element	TGA-element	TGACG-motif
*At1g47128*	1		6	3	1	–	–	1	2	–	6
*At5g60360*	4	–	3	1	3	–	1	–	1	–	3
*At4g16190*	–	–	1	7	–	–	–	–	–	1	1
*At4g14540*	8	2	2	–	–	–	1	1	–	1	2
*At5g54630*	3	–	2	1	2	1	–	–	–	2	2
*At5g43060*	2	1	–	2	2	–	–	–	1	–	–
*At5g45890*	–	–	–	1	–	–	–	–	–	–	–
*At4g39090*	1	–	–	–	–	–	–	1	–	–	–
*At3g45310*	3	–	2	1	2	1	–	–	–	–	2
*At1g02300*	–	–	1	–	–	1	–	–	–	–	1
*At4g01610*	4	–	1	–	–	1	–	1	–	1	1
*At4g11320*	1	–	1	4	1	–	–	–	2	1	1

**Note:**

Abscisic acid: ABRE; jasmonic acid: G-box, CGTCA-motif and TGACG-motif; ethylene: ERE; salicylic acid: TCA-element; auxins: AuxRR-core and TGA-element; and gibberellic acid: GARE-motif, P-Box and TATC-Box.

### Expression analysis of Lsi homologs in tomato, potato and *Arabidopsis* and Si-regulated genes in *Arabidopsis*

We explored different databases to retrieve expression-profiling data on *Lsi* genes from pepper. Since we were unable to find information on the pepper genome, we took data from other species closely related to pepper such as tomato and potato, all three belonging to the plant family Solanaceae. When no information on species of the family Solanaceae was found, or no detailed information was available, then we took data from *Arabidopsis thaliana* as a dicot species. *Arabidopsis* has been the major plant model system for the last three decades, giving rise to tremendous advances in our understanding of plant physiology and metabolism. As a eudicot species, discoveries in *Arabidopsis* can be applicable to many other plant species (Chang, Bowman & Meyerowitz, 2016).

In our first search, we used the eFP Browser (Hawkins et al., 2017) to explore different tomato plant tissues: epidermis, collenchyma, vascular, parenchyma, endodermis, unopened flower bud, fully opened flower, leaves, roots, 1 cm fruits, 2 cm fruits, 3 cm fruits, mature green fruits and breaker fruits. In potato, we analyzed stamens, mature whole fruits, tubers, immature whole fruits, petals, roots, carpels, whole in vitro plants, young tubers, whole mature flowers, inside of fruit tissues (mesocarp and endocarp), stolons, sepals, tuber peel, shoot apex, whole flowers, tuber sprouts, leaves, petioles, shoots in vitro, whole leaves, whole petiole, stems, tuber cortex and tuber pith. In *Arabidopsis*, we analyzed dry seeds, imbibed seeds, stamens, cauline leaves, cotyledons, roots, entire rosette after transition to flowering, carpels, petals, sepals, stamen, pedicels, petiole, distal and proximal half in leaf, hypocotyl, rosette leaves, senescing leaf, inflorescence/transition/vegetative shoot apex, mature pollen and vegetative rosette. File SF8 contains additional data concerning the analysis for *Lsi1* genes, whereas File SF9 comprises those for *Lsi2* genes.

In order to validate our data analysis, we also considered a set of genes comprising an external group for each species analyzed. Such genes were chosen because they do not encode for Lsi channels, and are predominantly expressed in the aboveground parts of the plant. Instead, *Lsi1* and *Lsi2* genes are mainly expressed in roots (Vatansever et al., 2017), which distinguish them from the external group analyzed. In this external group, we took into consideration the *SlC2H2*, *SlCP3* and *SlRD19A* genes from tomato; the *StC2H2*, *StRD19A*, *StRD21A* and *StCP3* genes from potato; and the *C2H2* (*At5g54630*) and three cysteine proteases (*At4g16190*, *At5g60360* and *At1g47128*) genes from *Arabidopsis*. This set of external genes from *Arabidopsis* shared 60% identity with their corresponding homologs in tomato and potato. The transcriptional activity of these external genes from *Arabidopsis* has been well demonstrated in the aerial parts of the plants but not in roots (Balazadeh, Riaño-Pachón & Mueller-Roeber, 2008; Bhalerao et al., 2003) (File SF10). With this, we aimed to prove that the *Lsi* genes have a different expression pattern in plant tissues and differential responsiveness to phytohotmones through the analisis of their promoter regions, as compared to the set of external genes.

Data retrieved from the eFP Browser were normalized to percentage scale. Afterwards, data were graphed as heatmaps with the help of a script in the R software (R Core Team, 2016), using the packages “gplots” and “RColorBrewer”.

**Figure 7:**
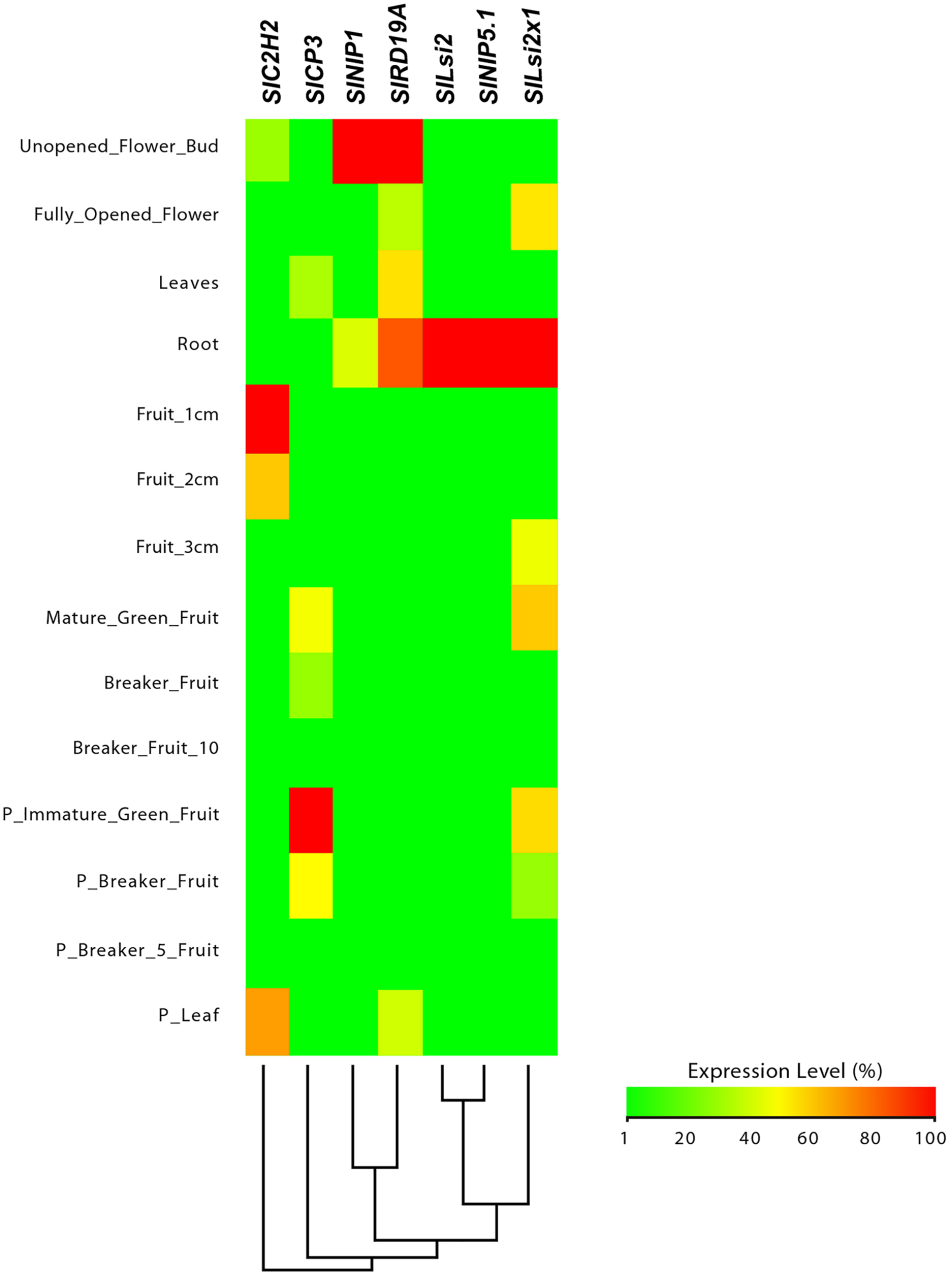
Tissue-specific expression patterns of tomato (*Solanum lycopersicum*) *SlLsi* genes homologs to pepper (*Capsicum annuum*) *CaLsi* genes. The *SlC2H2*, *SlCP3* and *SlRD19A* genes were considered as an external group for the purpose of this comparative analysis, since they do not encode Lsi channels. The heatmap was constructed with a script in the R v4.0.1 software using the functions of the packages “gplots” and “RColorBrewer”.

**Figure 8:**
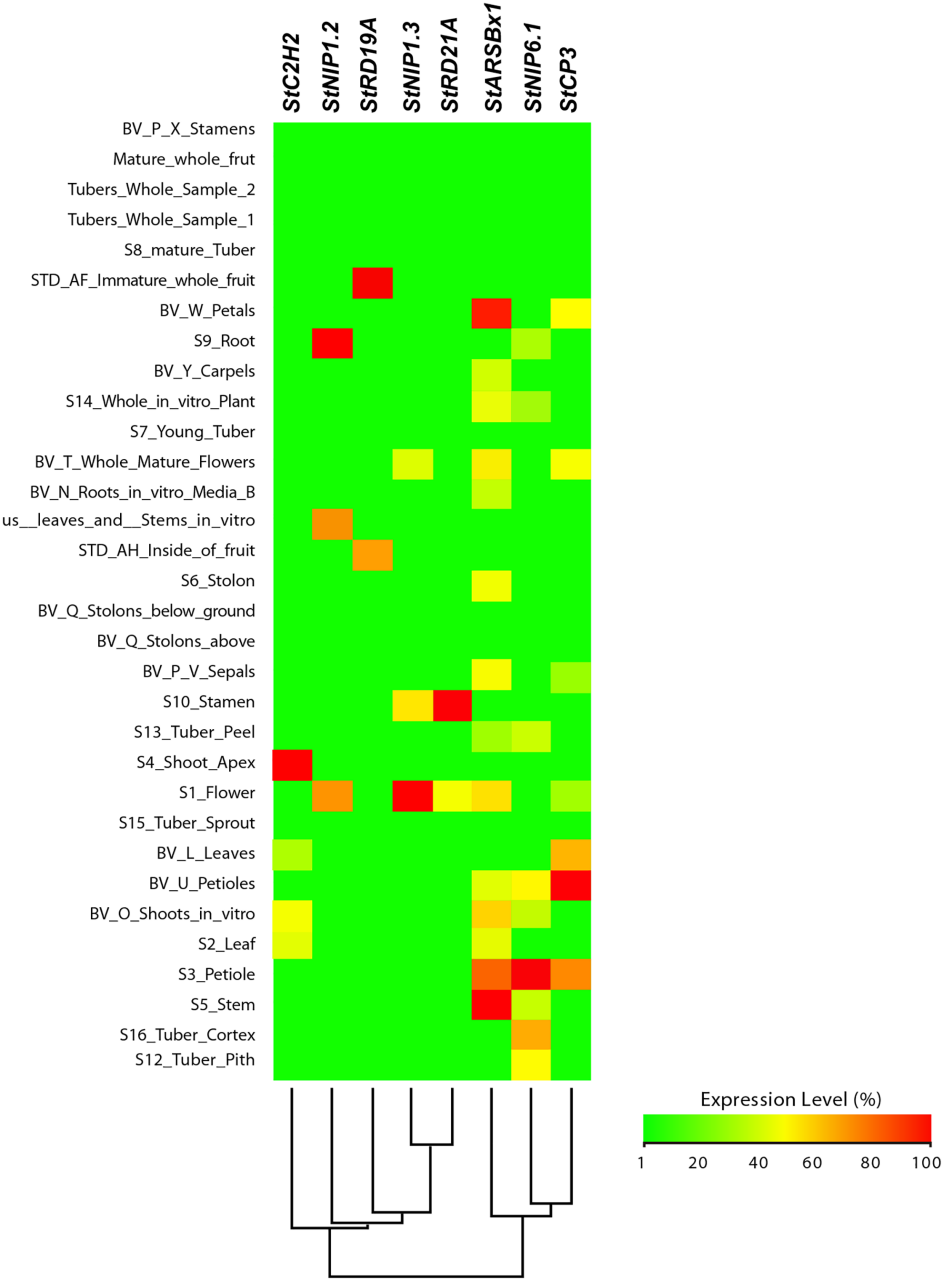
Tissue-specific expression pattern of potato (*Solanum tuberosum*) *StLsi* genes homologs to pepper (*Capsicum annuum*) *CaLsi* genes. The *StC2H2*, *StRD19A*, *StRD21A* and *StCP3* genes were considered as an external group for the purpose of this comparative analysis, since they do not encode Lsi channels. The heatmap was constructed with a script in the R v4.0.1 software using the functions of the packages “gplots” and “RColorBrewer”.

**Figure 9:**
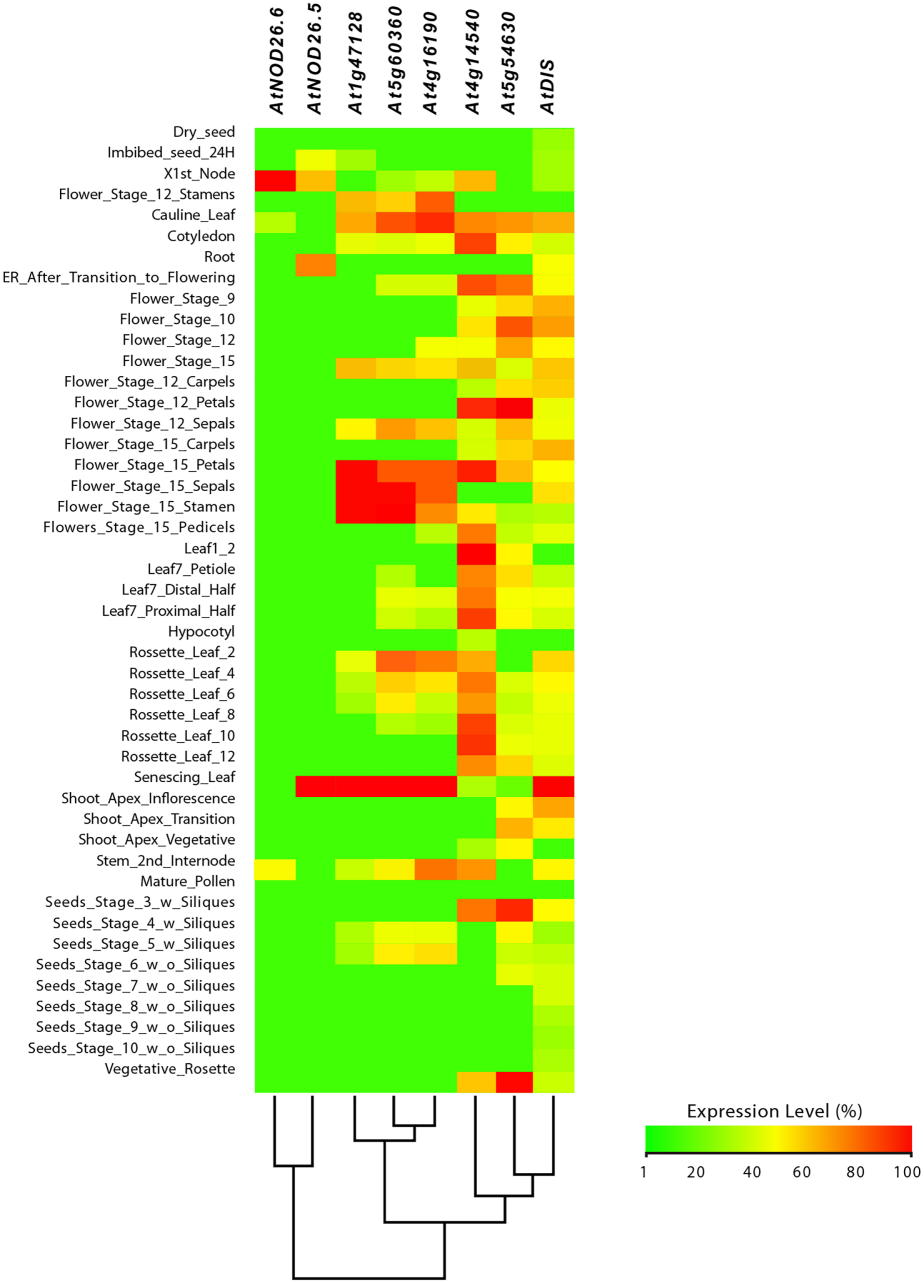
Tissue-specific expression pattern of *Arabidopsis* (*Arabidopsis thaliana*) *AtLsi* genes homologs to pepper (*Capsicum annuum*) *CaLsi* genes. The *C2H2*, *At4g16190*, *At5g60360* and *At1g47128* genes were considered as an external group for the purpose of this comparative analysis, since they do not encode Lsi channels. The heatmap was constructed with a script in the R v4.0.1 software using the functions of the packages “gplots” and “RColorBrewer”.

From this analysis, we detected different expression levels in the tomato *SlNIP1*, *SlNIP5.1*, *SlLsi2* and *SlLsi2x1* genes; in the potato *StNIP1.2*, *StNIP1.3*, *StNIP6.1* and *StARSBx1* genes; and in the *Arabidopsis AtNOD26.5*, *AtNOD26.6* and *AtDIS* genes (Table 1). In particular, in tomato, the *SlNIP5.1*, *SlLsi2* y *SlLsi2x1* genes displayed higher expression levels in roots as compared to leaves, flowers and fruits, while the *SlNIP1* genes was most expressed in roots and flowers (Fig. 7). In potato, the *StNIP1.2* gene exhibited the highest expression level in roots, though it was also expressed in leaves, while the expression of *StNIP1.3* was mainly detected in flowers, and *StNIP6.1* genes were mainly expressed in petioles of leaves, tubers and roots (Fig. 8). In *Arabidopsis*, the *AtNOD26.5* was mainly expressed in senescent leaves and roots; whereas *AtNOD26.6* had higher expression levels in the first node and internode in the stems; and *AtNOD26.5* was also expressed in senescent leaves and roots. The *AtDIS* gene was transcriptionally active in roots and senescent leaves, though it was also expressed in other tissues in a similar pattern as the external group of genes did (Fig. 9).

Regarding the set of genes of the external group in tomato (*SlC2H2*, *SlCP3* and *SlRD19A*), potato (*StC2H2*, *StRD19A*, *StRD21A* and *StCP3*), and *Arabidopsis* (*C2H2*, *At4g16190*, *At5g60360* and *At1g47128*), they all exhibited a similar expression pattern, mainly evident in the aboveground shoot system and repressed in the underground root system. The exception was the tomato *SlRD19A*, which was expressed both in shoot and root tissues (Fig. 7). This finding demonstrated that the set of genes of the external group were indeed differentially expressed as compared to the *Lsi* genes evaluated.

**Figure 10:**
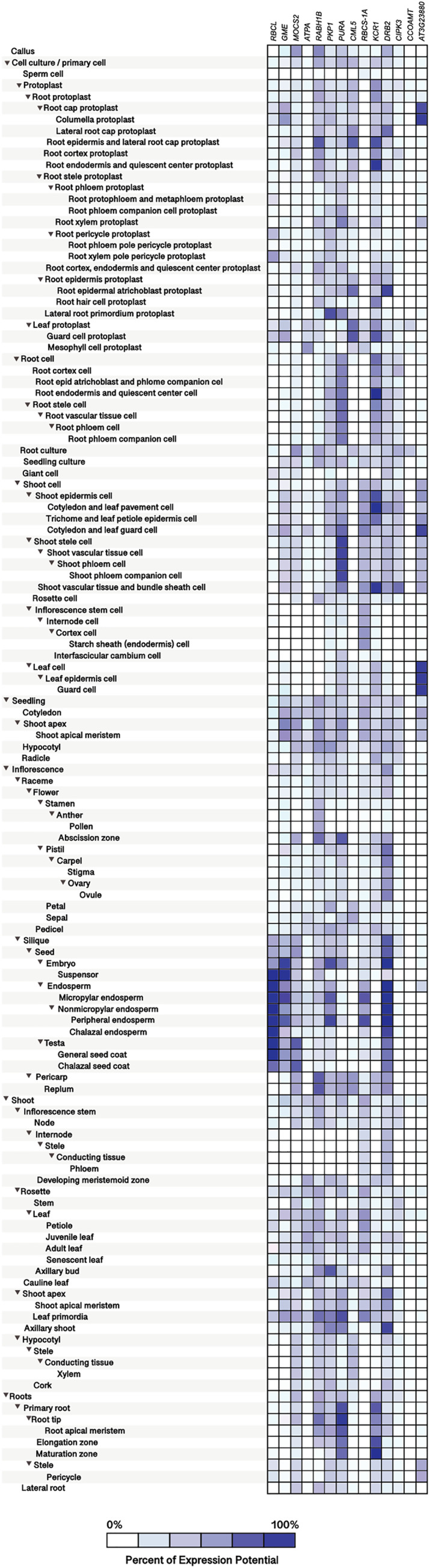
Tissue-specific expression patterns of homolog Si-regulated genes in *Arabidopsis* (*Arabidopsis thaliana*). All genes were selected based on their responsiveness to Si in *Capsicum annuum*. The specific expression by tissue in cell culture, seedling, inflorescence, shoot, and roots was obtained from the Genevestigator (https://genevestigator.com). Colors represent the intensity of the expression (percentage of expression potential), from white (0%) to dark blue (100%). More information regarding these experiments is included in File S11.

**Figure 11:**
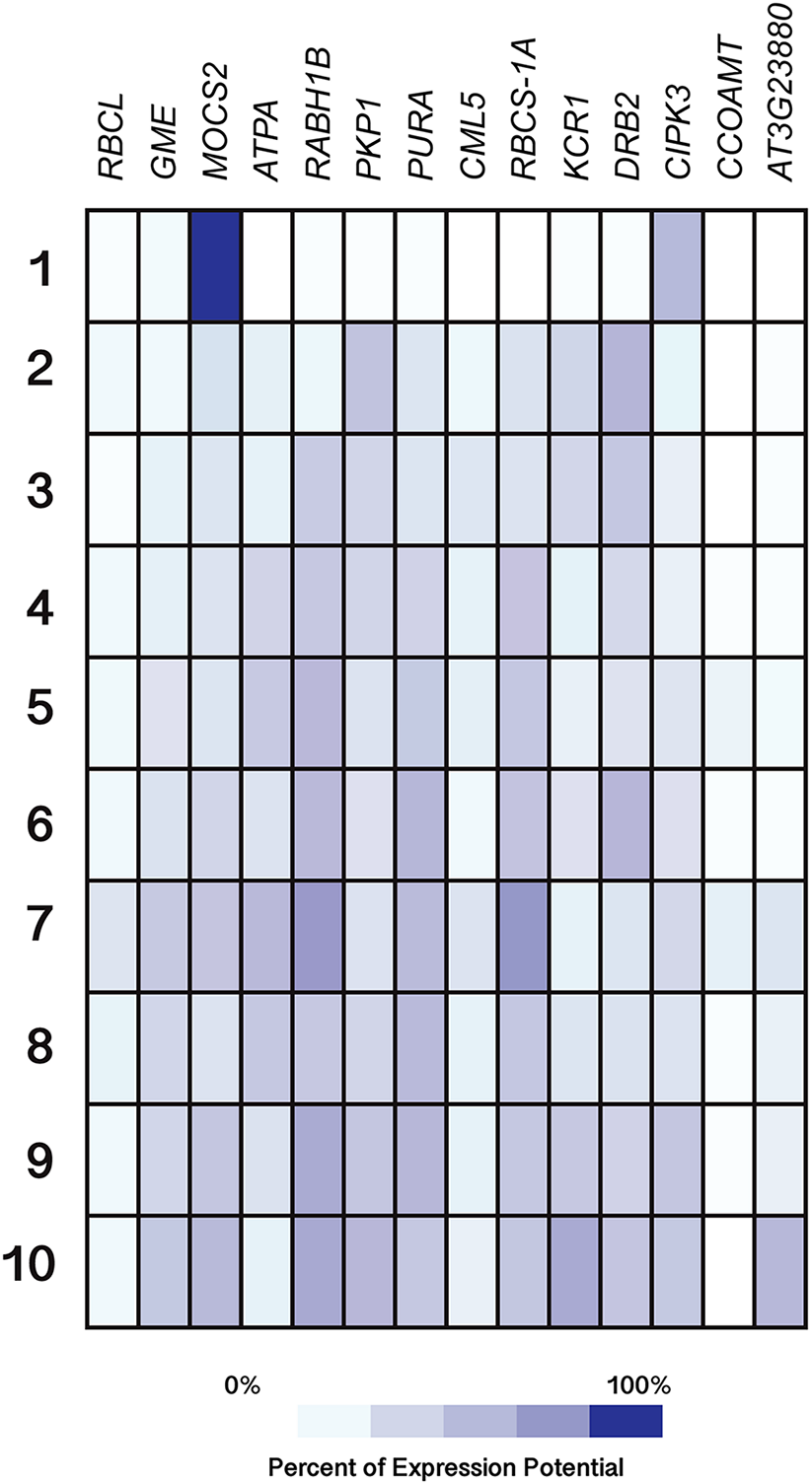
Expression patterns of homolog Si-regulated genes in *Arabidopsis* (*Arabidopsis thaliana*) from different developmental stages. All genes were selected based on their responsiveness to Si in *Capsicum annuum*. Germinated seeds (1), seedlings (2), young rosette leaves (3), developed rosette leaves (4), bolting (5), young flowers (6), developed flowers (7), flowers and siliques (8), mature siliques (9) and senescent plants (10). Data were retrieved from Genevestigator (https://genevestigator.com/gv/index.jsp). Colors represent the intensity of the expression (percentage of expression potential), from white (0%) to dark blue (100%). More information regarding these experiments is included in File S12.

**Figure 12:**
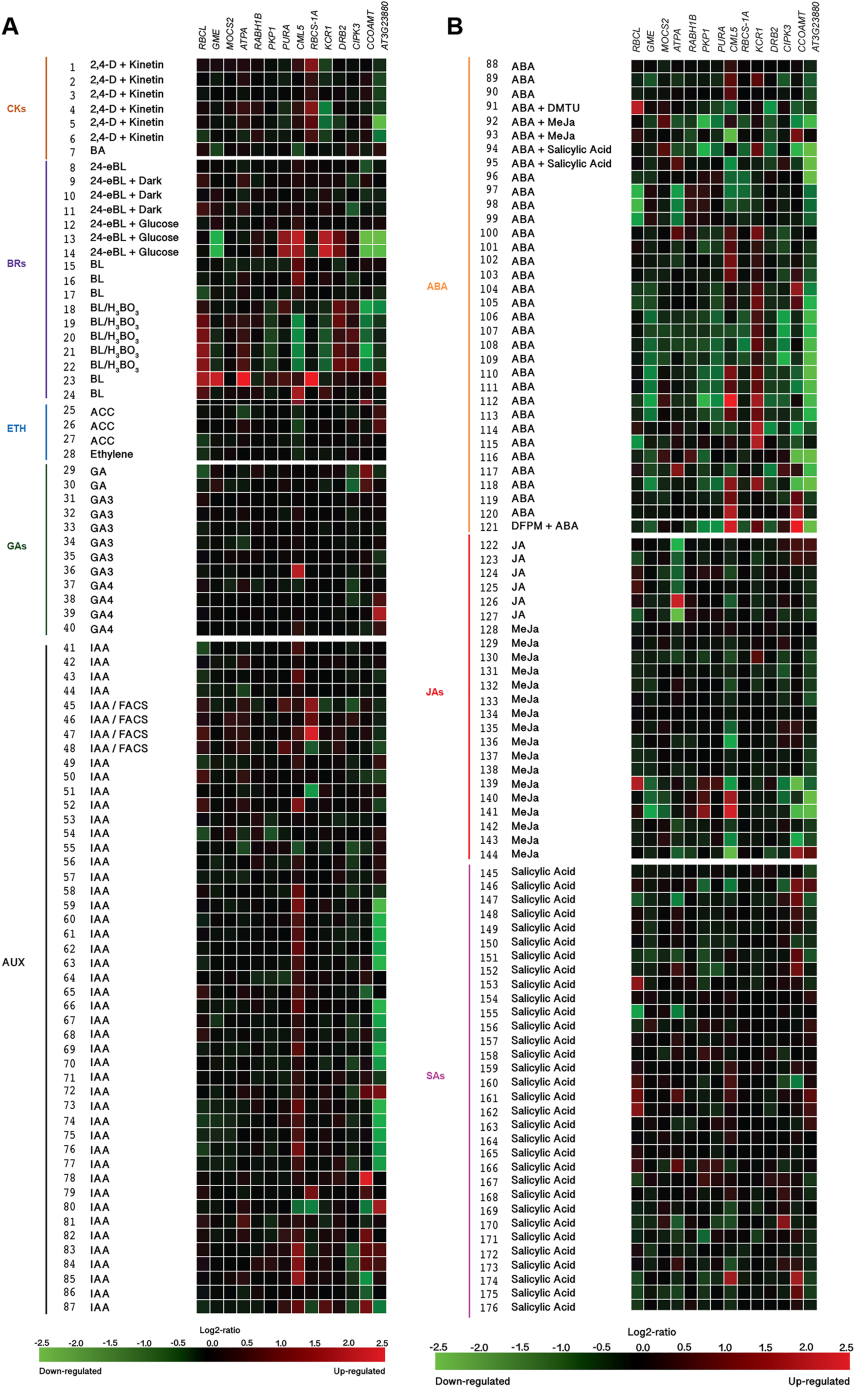
Expression patterns of homolog Si-regulated genes in *Aradidopsis* (*Arabidopsis thaliana)* in response to phytohormones or phytohormone precursors. (A) Responses to Cytokinins (CKs), Brassinosteroids (BRs), Ethylene (ETH), Gibberellins (GAs), and Auxins (AUX). (B) Responses to Abscisic acid (ABA), Jasmonates (JA), and Salicylic acid (SA). Expression data were retrieved from the Genevestigator (https://genevestigator.com). Color saturation corresponds to the degree of up-regulation (red) and down-regulation (green) of gene expression in the specified conditions. Expression changes that were assumed to be of little significance were colored in black. Experiments 1–7: Cytokinins (CKs; 2,4-D: 2,4-dichlorophenoxy acetic acid; BA: 6-Benzyl adenine); 8–24 Brassinosteroids (BRs: 24-BL, 24-Epibrassinolide; BL: Brassinolide); 25–28: Ethylene (ETH; ACC: 1-aminocyclopropane-1-carboxylic acid; ETH: Ethylene); 29–40: Gibberellins (GAs; GA3: Gibberellic acid 3; GA4: Gibberellic acid 4); 41–87: Auxins (AUX; IAA: Indole-3-acetic acid); 88–121: Abscisic acid (ABA); 122–144: Jasmonates (JA: Jasmonic acid; MeJA: Methyl-jasmonate); 145–176: Salicylic acid (SA). More information regarding these experiments is included in File S13.

Furthermore, expression-profiling data on the Si-regulated genes reported by Manivannan et al. (2016) were retrieved from Genevestigator (https://genevestigator.com/gv/index.jsp). Since this platform does not contain data on the pepper genome, expression-profiling analysis was based on *Arabidopsis thaliana* homologs to Si-regulated genes previously reported. Identification of homolog genes was carried out using the BlastP program (https://blast.ncbi.nlm.nih.gov/Blast.cgi?PAGE=Proteins). From the aforementioned 40 Si-regulated genes (Table 5), we were able to retrieve expression data of 14. Expression patterns of the corresponding genes were analyzed according to different plant tissues (Fig. 10; File SF11), development stages (Fig. 11; File SF12) and phytohormones (Fig. 12; File SF13).

**Table 5:** List of Si-regulated proteins found in different plant species and their homologs in *Arabidopsis* (*Arabidopsis thaliana*).

Si-regulated proteins		Homolog proteins in *Arabidopsis thaliana*			
Accession number	Organism	Accession number	Coverage (%)	Identity (%)	Gene name
XP_004249273	*Solanum lycopersicum*	NP_191320.1	89	83.88	*PURA*
XP_008793948	*Phoenix dactylifera*	OAP08906.1	99	51.36	–
XP_010243911	*Nelumbo nucifera*	–	–	–	–
XP_010655136	*Vitis vinifera*	NP_001322422.1	100	79.63	–
AHL68475	*Androcymbium* cf. capense Chacon 20	NP_051067.1	100	95.16	*RBCL*
KHG29665	*Gossypium arboreum*	–	–	–	–
XP_009393059	*Musa acuminata* subsp. *malaccensis*	NP_198236.1	100	90.79	*GME*
XP_003058724	*Micromonas pusilla*	–	–	–	–
XP_006647478.1	*Oryza brachyantha*	NP_565996	97	55.84	*CML5*
EMT25898	*Aegilops tauschii*	NP_176880.1	94	66.46	*RBCS-1A*
EMS51364	*Triticum urartu*	OAP06165.1	62	37.5	–
XP_009398204	*Musa acuminata* subsp. *malaccensis*	–	–	–	–
XP_010517956	*Camelina sativa*	NP_181904.1	97	92,78	*MOCS2*
XP_008677250	*Zea mays*	AAC49085.1	88	44.00	–
XP_010046336	*Eucalyptus grandis*	AAM90865.1	88	25.46	–
XP_004951624	*Setaria italica*	–	–	–	–
AFB70663	*Weingartia kargliana*	ANW47827.1	99	81.20	–
AEJ73201	*Nandina domestica*	–	–	–	–
XP_009588622	*Nicotiana tomentosiformis*	NP_176074.2	100	48.31	–
XP_004979712	*Setaria italica*	ABF00984.1	90	27.90	–
ABS87384	*Lactuca sativa*	NP_566720.1	90	85.93	*PKP1*
BAF01042	*Arabidopsis thaliana*	BAF01042.1	100	100.00	–
KIY92373	*Monoraphidium neglectum*	AAB60303.1	79	76.61	–
AAR08850	*Vitis amurensis*	–	–	–	–
AAL83898	*Zea mays*	NP_564905.1	91	44.21	*KCR1*
AAF72909	*Medicago sativa*	NP_001331467	100	34.18	–
AIF71068	*Actiniopteris semiflabellata*	NP_051044.1	100	90.10	*ATPA*
CAC87838	*Nicotiana tabacum*	–	–	–	–
XP_009147561	*Brassica rapa*	NP_001031521.1	93	43.55	–
XP_012064817	*Jatropha curcas*	NP_565672.1	72	66.58	*DRB2*
XP_006450493	*Citrus clementina*	–	–	–	–
Q2RAX3	*Oryza sativa* subsp. *japonica*	NP_850095.2	99	75.17	*CIPK3*
XP_009588623	*Nicotiana tomentosiformis*	–	–	–	–
BAB40826	*Zea mays*	BAB40828.1	100	86.71	–
KHG25806	*Gossypium arboreum*	–	–	–	–
AIU48281	*Illicium henryi*	AIU48266.1	100	74.69	–
AKL82244	*Rosa soulieana*	BAD94800.1	100	90.00	–
KHN40347	*Glycine soja*	NP_974104.1	88	57.30	*CCOAMT*
XP_006485364	*Citrus sinensis*	NP_181989.1	94	95.65	*RABH1B*
XP_003598507	*Medicago truncatula*	NP_189030.1	94	28.91	*AT3G23880*

**Note:**

Accession numbers of Si-regulated genes were as described by Manivannan et al. (2016). Further details of this list are described in File SF7.

The homolog genes in *Arabidopsis thaliana* were expressed in most tissues analyzed, although at different levels. Protoplasts exhibited higher expression levels of the Ras-related small GTP-binding family protein (*RABH1B*; NP_181989.1), Calcium-binding EF-hand family protein (*CML5*; NP_565996), and Beta-ketoacyl reductase 1 (*KCR1*; NP_564905.1). In root protoplasts, transcripts of GDP-mannose-3’, 5’-epimerase (*GME*; NP_198236.1), *RABH1B*, *CML5*, *KCR1*, dsRNA-binding protein 2 (*DRB2*; NP_565672.1), and F-box and associated interaction domains-containing protein (*AT3G23880*; NP_189030.1) were more abundant. In leaf protoplasts, expression levels of the genes *CML5* and Ribulose bisphosphate carboxylase small chain 1A (*RBCS-1A*; NP_176880.1) were more notable. In root cells, the genes Adenylosuccinate synthase (*PURA*; NP_191320.1) and *KCR1* were highly expressed, while in shoot cells the genes *PURA*, *RBCS-1A*, *KCR1*, CBL-interacting protein kinase 3 (*CIPK3*; NP_850095.2), and *AT3G23880* exhibited higher transcript levels as compared to the rest of the genes analyzed. In leaves, the gene *AT3G23880* was notably expressed. In the abscission zone of inflorescences, high expression of the genes molybdopterin biosynthesis MoaE protein (*MOCS2*; NP_181904.1), *RABH1B* and *PURA* was detected. In siliques and seeds, the genes Ribulose bisphosphate carboxylase small chain 1A (*RBCS-1A*; NP_176880.1), *GME*, Pyruvate kinase family protein (*PKP1*; NP_566720.1), *RBCS-1A* and *DRB2* were highly expressed. In senescent plants, we detected high expression of *PKP1* and *DRB2*, while in shoot apex and axillary shoot the gene *DRB2* was also highly expressed. Finally, in roots, the genes *PURA* and *KCR1* displayed higher expression as compared to the rest of the genes evaluated (Fig. 10).

Regarding development stages, higher levels of expression were observed in the gene *MOCS2* in germinated seeds as compared to other tissues analyzed, while the genes *RABH1B* and *RBCS-1A* were moderately expressed in developed flowers. The rest of the genes analyzed displayed very low expression levels or were indeed repressed in the tissues evaluated (Fig. 11).

Exposure to silicon has been correlated with changes in plant hormonal balance. In particular, a positive correlation between Si exposure and active cytokinin concentrations has been reported (Markovich et al., 2017). Our in silico analysis based on the Genevestigator platform demonstrated that Si-regulated genes identified in *Arabidopsis thaliana* (homologs to those identified in *Capsicum annuum*) are responsive to phytohormones and phytohormone precursors (Fig. 12). For instance, the combination of 2,4-dichlorophenoxyacetic acid (2,4-D) and kinetin induced the expression of *RBCS-1A* but repressed that of *KCR1* and *AT3G23880*. The cytokinin 6-benzylaminopurine (BA) downregulated the expression of *GME* and *CML5*. The expression of the genes caffeoyl-CoA 3-O-methyltransferase (*CCOAMT*; NP_974104.1), *CIPK3* and *AT3G23880* was repressed by 24-epibrassinolide (24 eBL), while *PKP1*, *PURA*, *KCR1* and *DRB2* were induced by this brassinosteroid. Ribulose-1,5-bisphosphate carboxylase/oxygenase large subunit (*RBCL*; NP_051067.1), *GME*, *ATPA*, *PKP1*, *PURA*, *CML5*, *RBCS-1A* and *AT3G23880* were transcriptionally induced by brassinolide (BL) as compared to mock treated seedlings, while when comparing to untreated cell cultures, BL reduced the expression of *CML5*, *KCR1* and *CCOAMT*. The ethylene precursor 1-aminocyclopropane-1-carboxylate (ACC) and ethylene itself had low impact on the expression of the genes under study, though a slight induction of *AT3G23880* and a very low repression of *RBCL*, *ATPA* and *CML5* were observed. Gibberellic acid (GA) induced the expression of *CML5* and *AT3G23880*, while that of *CIPK3* was slightly repressed. The auxin indole-3-acetic acid (IAA) notably induced the expression of *CML5* and *CCOAM*. Interestingly, IAA both induced and repressed the expression of *RBCS-1A* and *AT3G23880*, depending on the experimental conditions imposed: the combination of IAAwith fatty acid-amino acid conjugates (FACS) induced the expression of *RBCS-1A* in roots, whereas it was repressed in seedlings. As for *AT3G23880*, its expression was induced in leaves, while in seedlings it was repressed in response to IAA. Abscisic acid (ABA) also exhibited contrasting effects on gene expression depending on the experimental conditions tested. For instance, in combination with *N*,*N*-dimethylthiourea (DMTU, a H_2_O_2_ scavenger), ABA induced the expression of *RBCL* in cell suspensions, while *DRB2* was repressed. Conversely, in seeds, ABA downregulated the expression of *RBCL* and *ATPA*. ABA also upregulated the expression of *CML5* and *KCR1*, but downregulated that of *AT3G23880*. Similarly, jasmonic acid (JA), methyl jasmonate (MeJa) and salicylic acid triggered contradictory responses in most genes evaluated.

## Discussion

### Silicon is mostly concentrated in roots, and little is transported to stems and leaves

In our study, Si concentration in roots was significantly higher in Si-treated plants. Indeed, the higher the Si supply, the greater the Si concentration found in tissues analyzed. In stems, no differences among treatments were observed, while in roots, Si-treated plants displayed higher Si concentrations, but all of them were lower than those observed in stems and roots. Thus, pepper plants retain Si in the roots while only small amounts of this element are transported to the leaves, which is a biologically limiting factor that can be responsible for many plants not responding to Si applications (Deshmukh & Bélanger, 2016). In tomato, SlLsi1 influx channel transports Si in the roots, while two Lsi2-like proteins did not show efflux transport activity for Si, thus Si imported by SlLsi1 could not be exported from roots to shoots (Sun et al., 2020). Nevertheless, tomato has been shown to increase productivity in response to Si applications, which results in more erect leaves, improved photosynthetic activity, and higher concentrations of chlorophylls (Pereira, Vitti & Korndorfer, 2003; Braga et al., 2009; Marodin et al., 2014). If Si is not transported from the roots to the shoots, and even though stimulatory effects are observed, then the Si deposition in the apoplast could trigger the beneficial effects observed, with hormetic dose-response effects, which is in agreement with the apoplastic obstruction hypothesis postulated by Coskun et al. (2019a).

Si-accumulator species such as rice can accumulate Si in the aerial part (including grains and straw) by increasing the applied dose of Si (Cuong et al., 2017). Similarly, wheat can transfer approximately 90% of the Si from the roots to the shoots, and the Si concentration in roots displays lower levels as compared to aerial parts (Abdel Latef & Tran, 2016).

There is a wide variation in the concentration of Si in the aerial part in different plant species, ranging from 0.1 to 10% of dry biomass weight, and these differences have been attributed to the capacity of roots to absorb Si and the internal cellular mechanisms to mobilize it within the plant (Takahashi, Ma & Miyake, 1990; Song et al., 2009; Ma & Yamaji, 2015; Sahebi et al., 2015). Within the angiosperms, the families Cyperaceae, Poaceae and Balsaminaceae show high accumulation of Si (i.e., more than 4% Si on a dry matter basis), while the families Cucurbitaceae, Fabaceae, Urticaceae and Commelinaceae accumulate moderate levels of this element (i.e., 2–4% Si on a dry matter basis). Most plant species, particularly dicots, accumulate Si at a relatively low level. The difference in Si accumulation among plant genotypes can be explained, at least partially, by the differences in the Si uptake ability of the roots attributed to the existence, density and localization of Si channels (Ma & Takahashi, 2002; Hodson et al., 2005; Ma & Yamaji, 2006). Consequently, agricultural crops may display marked differences with regard to their capacity to absorb Si and transport it from the roots to upper parts of the plant (Marschner, 2012; Tubaña & Heckman, 2015; Tubaña, Babu & Datnoff, 2016).

As aforementioned, the genotypic difference in Si accumulation results from the differences in abundance of Lsi channels (both Lsi1 and Lsi2) in roots (Ma, 2010) involved in the uptake and distribution of Si within the plant (Ma et al., 2006; Ma et al., 2007; Ma & Yamaji, 2015; Sasaki, Yamaji & Ma, 2016). These two groups of channels are localized to the plasma membrane. Among plant species, the Lsi channels exhibit divergent abundance, expression patterns and localization within cells and tissues, which concomitantly result in different capacities of Si accumulation (Ma & Yamaji, 2015). A recently developed model of Si dynamics in rice reproduced the gradual decrease and diurnal variation of channel gene expression levels, demonstrating that a considerable reduction in the expression of Si channel genes during the night increases investment efficiency (Sakurai et al., 2017).

It is worthwhile mentioning that a genotypic variation of Si concentration in shoot has been found both within and among plant species, although the former variation is usually much lower (Deren, 2001; Ma et al., 2006; Ali, 2009; Broadley et al., 2012). The distinct capacities of Si accumulation in the plant kingdom is attributed to the activity of Lsi channels (Yan et al., 2018). Indeed, crop species such as tomato, potato and flax (*Linum usitatissimum*), which exhibit Si contents between 0.2% and 0.5% on a dry basis (thus classified as poor Si accumulators), are devoid of functional Lsi2 channels (Shivaraj et al., 2017).

Once within the plants, Si can trigger numerous beneficial effects, including an increase in water absorption through the roots, maintenance of nutrient balance, a decrease in water loss through the leaves, and stimulation of a higher photosynthetic rate (Zhu & Gong, 2014). Furthermore, Si can induce resistance or tolerance mechanisms in response to stress conditions through the regulation of genes involved in photosynthesis, water absorption and use, transcription, secondary metabolism, and polyamines biosynthesis (Manivannan & Ahn, 2017; Abdel-Haliem et al., 2017).

Nevertheless, such statements are currently being challenged since there is limited genetic variability for Si accumulation, at least within the germplasm studied (Ouellette et al., 2017). Thus, it has been well established that Si accumulation displays limited within-species variability, representing a genetic trait tightly regulated by the expression and activity of Lsi proteins (Deshmukh et al., 2015). Interestingly, Si is not translocated to strawberry fruit, thereby eliminating concerns about Si potentially affecting fruit firmness or quality (Ouellette et al., 2017).

Alhough the beneficial effects of Si have been widely reported, the mode of action and biological function of Si in plants have been the subject of continuing scientific debate (Coskun et al., 2019b; Exley & Guerriero, 2019).

### The pepper genome contains putative Lsi channels potencially involved in Si uptake and transport

In the family Solanaceae, to which crop species such as pepper, tomato, potato, tobacco (*Nicotiana tabacum*), and eggplant (*Solanum melongena*) belong, putative Lsi channels have been identified, as described below.

The pepper genome contains at least 16 influx channels (named CaLsi1 for *Capsicum annuum* Low silicon channel 1) and three efflux ones (CaLsi2) involved in Si uptake and transport within the plant cell. Importantly, all *Lsi1* and *Lsi2* isogenes found in the pepper genome are transcriptionally active, as was evident from the EST/mRNA/cDNA data analysis we performed (Table 1). Nevertheless, the low abundance of Lsi2 genes (only three putative genes found), suggest a poor capacity of pepper to translocate Si from roots to shoots.

The Lsi1 proteins mediate the transport of Si across the plasma membrane between the external solution and the plant cell. Their length varies between 191 and 344 amino acid residues, with 20.59 and 37.35 kDa in molecular mass, respectively. They belong to the Nodulin-26-like intrinsic protein (NIP) III subgroup of the aquaporin membrane protein family and display distinct selectivity (Ma & Yamaji, 2015), facilitating the passive transport of water as well as other uncharged solutes such as glycerol (Wallace & Roberts, 2005), ammonia and urea (Beitz et al., 2006), boric acid (Takano et al., 2006), lactic acid (Choi & Roberts, 2007), selenite (Zhao et al., 2010), arsenite and silicic acid (Mitani-Ueno et al., 2011). Mechanisms responsible for the specificity of transport substrates between different NIP subgroups have been identified, revealing that the serine residue (S) at the H5 position of the aromatic/arginine (ar/R) pore of NIP III proteins determines the permeability to Si and B, but not to arsenite (Mitani-Ueno et al., 2011). Other nightshade species such as tomato and potato also contain NIP III representatives (Ma & Yamaji, 2015). Just recently, a functional Lsi1 channel, NtNIP2;1, was identified and characterized in tobacco, which is a low Si accumulator belonging to the family Solanaceae as well (Zellner et al., 2019). Moreover, in tomato, the protein SlLsi1 was found to be transcriptionally and enzymatically active in the roots, but did not alter the Si concentration in the shoots. Instead, the two SlLsi2-like proteins identified did not transport Si (Sun et al., 2020). These findings may help explain that the low Si concentrations found in the upper parts of tomato plants are due to the absence of functional Si efflux channels. Nevertheless, tomato positively responds to silicon applications, as proven in various studies (Ali et al., 2018; Khan et al., 2019; Wu et al., 2015; Zhang et al., 2018a). From our search, we discovered that other dicotyledonous species such as cucumber (*Cucumis sativus*), soybean, strawberry and wild strawberry (*Fragaria vesca*) also contain Lsi1 representatives in their corresponding genomes.

The Lsi2 efflux channels are responsible for the transport of Si out of the cell within the plant (Ma & Yamaji, 2015). They belong to the citrate transporter family and demonstrate neutral or slightly basic properties. Their length fluctuates between 491 and 532 amino acids, with a molecular mass between 52.9 and 57.89 kDa, respectively. They are predicted to localize in the plasma membrane with 10–11 putative TMDs, having extracellular N- and extracellular/cytoplasmic C-terminal regions. Lsi2 transcripts contained the 2–4 exons but mostly with 2–3. All identified Lsi2 homologs showed the high *e*-value in blastp search, implying their other roles in Si transport because they were also highly preserved even in low Si-accumulators (File SF1).

The coordinated activity of Lsi1 and Lsi2 allow Si to enter the plant and to be loaded into the xylem, thus facilitating root-to-shoot translocation (Coskun et al., 2019a). Consequently, the abundance and activity of Lsi2 channels determine the capacity of the plant to move Si from the root to the shoot. In our case, the pepper genome contains 16 putative Lsi1 and three putative Lsi2 channels. Therefore, one would expect that pepper is efficient at absorbing Si from the soil solution, but deficient at translocating it from the root to the shoot, as observed in our experimental data.

### Lsi channels display responsiveness to phytohormones

Plant growth stimulation triggered by Si can be (at least in part) explained by the Si-mediated action on phytohormone biosynthesis (Luyckx et al., 2017b) and metabolism (Haddad et al., 2019). If Si-responsive genes were also regulated by phytohormones, then a possible convergence between the signaling pathways mediated by phytohormones and the role of Si in growth regulation could be elicited. Indeed, our promoter analysis revealed that both *Lsi1* and *Lsi2* genes exhibit *cis*-acting elements and motifs responsive to abscisic acid (ABA), jasmonic acid (JA), ethylene (ETH), salicylic acid (SA), auxins (AUX), and gibberellic acid (GA) (Table 2). Likewise, Si-regulated genes also contain *cis*-acting elements in their promoter regions responsive to phytohormones and phytohormone precursors (Table 3). It is worthwhile noting that in the presence of toxic levels of cadmium and copper, rice plants treated with Si reduced JA and SA concentrations, while ABA was exponentially activated with increasing stress periods (Kim et al., 2014). Since ABA exerts an antagonistic effect on JA/SA biosynthesis (Luyckx et al., 2017b), the aforementioned responses could be the result of intrinsic homeostatic mechanisms triggered by Si. Moreover, short-term Si application reduced endogenous JA in rice plants exposed to wounding stress (Kim et al., 2011), while soybean plants exposed to salt stress (80 mM NaCl) in hydroponics increased gibberellin concentrations when treated with Si (Lee et al., 2010). Si has also been demonstrated to improve the biosynthesis of SA, JA and ETH in the presence of diverse pathogens (Fauteux et al., 2005, 2006; Ghareeb et al., 2011). In rice, Si amendment differently regulated the expression of 221 genes, including genes controlling ethylene signal transduction pathways (Brunings et al., 2009). Si increases cytokinin biosynthesis in *Arabidopsis* and sorghum (*Sorghum bicolor*) (Markovich et al., 2017). In licorice (*Glycyrrhiza uralensis*) exposed to salt stress, Si improves phytohormonal balance, especially that of indole-3-acetic acid (IAA) and GA (Zhang et al., 2018b). Since phytohormones influence vital biological mechanisms within the plant, interactions among them are of paramount importance to regulate numerous processes involved in plant growth, development, and response to biotic and abiotic stresses. Currently, we are carrying out biochemical, physiological and molecular approaches aimed at determining variations of main phytohormones in response to Si and other beneficial elements in crop plants such as rice, maize, pepper and tomato.

### Homolog *Lsi* genes are mainly expressed in root tissues in tomato, potato and *Arabidopsis*

Information on the expression pattern of *Lsi* genes in pepper was not available in the platforms we explored. Therefore, we analized expression data on tomato and potato, which also belong to the family Solanaceae, and the eFP Browser contained information on them but not on pepper. When no data on either tomato or potato were found, we collected data from *Arabidopsis thaliana* as a dicot species. From our search, we found that the tomato *SlNIP5.1*, *SlLsi2* and *SlLsi2.1* genes were mainly expressed in roots, whereas *SlNIP1* was transcriptionally active in roots and leaves. In potato roots and tubers, high expression of the *StNIP1.2*, *StNIP1.3* and *StNIP6.1* genes was found. During the seedling stage of *Arabidopsis*, the *AtNOD26.5* and *AtNOD26.6* genes were expressed in all tisses, whereas *AtNOD26.5* is more expressed in root tissues. The *AtDIS* gene was also mainly expressed in roots and stems of *Arabidopsis* seedlings, whereas in plants it was mainly expressed in leaves. The external group of genes was mainly expressed in the shoot system and repressed in the root system. This evidence confirms that the *Lsi* genes are mainly expressed in root tisses, which coincides with other reports (Deshmukh et al., 2013; Vatansever et al., 2017; Sun et al., 2020).

### Si-regulated genes involved in major physiological processes also exhibit phytohormone responsiveness and are expressed in different tissues and developmental stages in *Arabidopsis*

There is plenty of evidence demonstrating the beneficial effects of Si on vital physiological plant processes. In canola (*Brassica napus*), Si differentially regulated genes involved in different metabolic pathways, especially in cell wall synthesis, phytohormone metabolism, and stress response, leading to a better adaptation to challenging environments (Haddad et al., 2019). In pepper, Si affected the expression of key enzymes such as Adenylosuccinate synthetase (involved in nucleotide biosynthesis); E3 ubiquitin-protein ligase PUB23-like (negative regulator of water stress responses); Zinc finger protein 160-like (transcriptional regulation); Vacuolar protein sorting-associated protein 53 A isoform X2 (endocytic recycling); Ribulose-1,5-bisphosphate carboxylase/oxygenase large subunit (key photosynthetic enzyme); GDP-mannose 3,5-epimerase 2-like (part of vitamin C biosynthesis in plants); Nucleoporin-like protein (component of the nuclear pore complexes); Calcium-binding protein CML17-like (root hair elongation); Oxygen-evolving enhancer protein 3-1, chloroplastic-like (photosystem II assembly/stability and photoautotrophic growth); Molybdopterin synthase catalytic subunit-like (conversion of precursor Z into molybdopterin); MADS-box transcription factor 26 isoform X2 (positive regulation of transcription of RNA polymerase II); Disease resistance protein RPS2-like (pathogen defense responses); Mediator of RNA polymerase II transcription subunit 11-like (regulation of the RNA polymerase II transcription machinery); Mitochondrial calcium uniporter regulatory subunit MCUb-like isoform (calcium uptake into the mitochondrion); Disease resistance RPP13-like protein 3-like (defense responses); F-box only protein 8-like (signal transduction); Double-stranded RNA-binding protein (nucleocytoplasmic transport), Tudor-like RNA-binding protein (RNA binding); Calcium uniporter (calcium transport); Ras-related protein RABH1b-like (protein transport and cell growth); and F-box/kelch-repeat protein (phenylpropanoid metabolic process) (Manivannan et al., 2016). Some of the corresponding homolog genes found in *Arabidopsis thaliana* demonstrated differential expression among tissues, developmental stages and hormone treatments. Furthermore, the promoter regions of genes encoding some of these enzymes exhibit *cis*-acting elements responsive to phytohormones, pointing to a role of Si in the regulation of growth mediated by phytohormones.

Just recently, Coskun et al. (2019a) proposed a working model named ‘apoplastic obstruction hypothesis’, which established that Si deposits in the apoplast both interferes with and promotes a number of biological events leading to its beneficial role, with important cascading effects on plant morphology and physiology in a hormetic manner. Indeed, orthosilicic acid (the only Si available form for plants) can be soluble in water up to ca. 2 mM at 25 °C, above which polymerization into silica (SiO_2_) gel begins to occur (Ma, Miyake & Takahashi, 2001), which can be toxic for plants (Exley, 2015; Montpetit et al., 2012). This may explain, at least in part, the negative effects of Si applied at higher levels in our previous study (Trejo-Téllez et al., 2020) and point to a role of Si in triggering hormesis in plants. Hormesis is a biphasic dose-response phenomenon triggered by an environmental agent, with low doses stimulating beneficial effects and high doses inhibitory or toxic effects, which has wide implications for risk assessment, plant stress biology, and agriculture (Agathokleous, Kitao & Calabrese, 2020). Therefore, since hormesis can also result in phenotypic changes and adaptation strategies in plants under challenging environments (Macias-Bobadilla et al., 2020), this phenomenon has the potential to enhance agricultural sustainability in a changing world (Agathokleous & Calabrese, 2019). Importantly, even under non-stressful environmental conditions, Si has been proven to stimulate plant growth and metabolism (Trejo-Téllez et al., 2020; Xu et al., 2020), though its beneficial effects are more evident under stress conditions (Ahanger et al., 2020; Bui, Duong & Nguyen, 2020; Mandlik et al., 2020; Singh et al., 2020; Vaculík et al., 2020).

## Conclusions

In pepper plants, Si concentrates mainly in the root, while only a small part is translocated to the stems and leaves.

We were able to identify *Lsi1* and *Lsi2* candidate genes in the *Capsicum annuum* genome. Importantly, they proved to be transcriptionally active. The higher number of Lsi1 than Lsi2 genes suggests that this species can indeed absorb Si in the root cells, but lacks the molecular mecanisms to translocate it to the shoot system. Molecular cloning of the Lsi2 channels should, hypothetically, shed light on their enzymatic activity and efficiency at translocating Si from roots to shoots.

Analysis of the promoter regions of *CaLsi1* and *CaLsi2* revealed the presence of *cis*-acting elements and motifs responsive to phytohormones.

Homolog Lsi channels found in tomato, potato and *Arabidopsis* display higher expression levels in roots as compared to other tissues analysed. Furthermore, *cis*-active elements responsive to phytohormones were more abundant in the promoter regions of these genes as compared to the set of genes of the external group evaluated, thereby providing new insights into the connection existing among Si, phytohormone signaling and stimulation of plant growth and metabolism.

Furthermore, Si-regulated genes in *Arabidopsis* (homologs to those found in pepper) were active in different tissues and developmental stages, and differentially regulated by phytohormones and phytohormone precursors such as 2,4-D, BA, 24-EBL, BL, ACC, GA, GA3, IAA, JA, MeJa and SA. These genes are involved in vital physiological processes including transcription, nucleotide biosynthesis, endocytic recycling, photosynthesis, carbon metabolism, nutrient metabolism, signal transduction, intracellular transport, cell growth, phenylpropanoid metabolism, and stress responses.

To the best of our knowledge, this study represents the first attempt to provide an in-depth evaluation correlating Si-driven responses and phytohormones in pepper, supported by in vivo and in silico data analyses.

## Supplemental Information

10.7717/peerj.10053/supp-1 Supplemental Information 1 Raw data: Effect of silicon on Si concentrations in different tissues of the pepper plant.

10.7717/peerj.10053/supp-2 Supplemental Information 2 Silicon transporters *Lsi1* and *Lsi2* genes identified in different plant species. *Cs: Cucumis sativus; Hv: Hordeum vulgare; Os: Oryza sativa; Sb: Sorghum bicolor; Ta: Triticum aestivum; Zm: Zea mays; Ea: Equisetum arvense; Gm: Glycine max; Fv: Fragaria vesca; Fa: Fragaria x ananassa*. Si-responsive genes according to Ouellette et al. (2017).

10.7717/peerj.10053/supp-3 Supplemental Information 3 Promoter sequences of pepper (*Capsicum annuum*) *Lsi* genes used to identify *cis*-acting elements and motifs involved in phytohormone responses.

10.7717/peerj.10053/supp-4 Supplemental Information 4 Promoter sequences of pepper (*Capsicum annuum*) Si-regulated genes according to Manivannan et al. (2016) used to identify *cis*-acting elements and motifs involved in phytohormone responses.

10.7717/peerj.10053/supp-5 Supplemental Information 5 Promoter sequences of the external group of genes used to identify *cis*-acting elements and motifs involved in phytohormone responses.

10.7717/peerj.10053/supp-6 Supplemental Information 6 List of *cis*-regulatory elements responsive to phytohormones found in the promoter regions of *Lsi1* and *Lsi2* genes in the pepper (*Capsicum annuum*) genome.

10.7717/peerj.10053/supp-7 Supplemental Information 7 List and characteristics of Si-regulated proteins reported by Manivannan et al. (2016) in pepper (*Capsicum annuum*)..

10.7717/peerj.10053/supp-8 Supplemental Information 8 Analysis of the 2000 bp nucleotide sequences upstream of the start codon of homolog Si-regulated genes of pepper (*Capsicum annuum*) reported by Mannivannan et al. (2016).

10.7717/peerj.10053/supp-9 Supplemental Information 9 Detailed description of experiments aimed at identifying expression patterns of homolog Lsi1 genes from tomato (*Solanum lycopersicum*), potato (*Solanum tuberosum*) and *Arabidopsis* (*Arabidopsis thaliana*) in different plant tissues. Data and figures were retrieved from the eFP Browser tool hosted by the Bio-Analytic Resource developed by the University of Toronto (http://bar.utoronto.ca/), from the following sources: Waese & Provart, 2016; Waese et al., 2017; Winter, 2007. Image credit: Nicholas J. Provart.

10.7717/peerj.10053/supp-10 Supplemental Information 10 Detailed description of experiments aimed at identifying expression patterns of homolog *Lsi2* genes from tomato (*Solanum lycopersicum*), potato (*Solanum tuberosum*) and *Arabidopsis* (*Arabidopsis thaliana*) in different plant tissues. Data and figures were retrieved from the eFP Browser tool hosted by the Bio-Analytic Resource developed by the University of Toronto (http://bar.utoronto.ca/), from the following sources: Waese & Provart, 2016; Waese et al., 2017; Winter, 2007. Image credit: Nicholas J. Provart.

10.7717/peerj.10053/supp-11 Supplemental Information 11 Detailed description of experiments aimed at identifying expression patterns of the set of external genes from tomato (*Solanum lycopersicum*), potato (*Solanum tuberosum*) and *Arabidopsis* (*Arabidopsis thaliana*) in different plant tissues. Data and figures were retrieved from the eFP Browser tool hosted by the Bio-Analytic Resource developed by the University of Toronto (http://bar.utoronto.ca/), from the following sources: Waese & Provart, 2016; Waese et al., 2017; Winter, 2007. Image credit: Nicholas J. Provart.

10.7717/peerj.10053/supp-12 Supplemental Information 12 Detailed description of experiments aimed at identifying tissue-specific expression patterns of homolog Si-regulated genes in *Arabidopsis thaliana*. Gene expression levels can be visualized in Fig. 10. Data were retrieved from the Genevestigator platform available at https://genevestigator.com/gv/ (Zimmermann et al., 2014).

10.7717/peerj.10053/supp-13 Supplemental Information 13 Detailed description of experiments aimed at identifying expression patterns of homolog Si-regulated genes in different developmental stages in *Arabidopsis thaliana*. Gene expression levels can be visualized in Fig. 11. Data were retrieved from the Genevestigator platform available at https://genevestigator.com/gv/ (Zimmermann et al., 2014).

10.7717/peerj.10053/supp-14 Supplemental Information 14 Detailed description of experiments aimed at identifying expression patterns of homolog Si-regulated genes in response to phytohormones and phytohormone precursos in *Arabidopsis thaliana*. Gene expression levels can be visualized in Fig. 12. Data were retrieved from the Genevestigator platform available at https://genevestigator.com/gv/ (Zimmermann et al., 2014).

## Additional Information and Declarations

### Data Availability

The following information was supplied regarding data availability: The raw measurements are available in the Supplemental Files.
