# Supplementary material for: Silicon flow from root to shoot in pepper: a comprehensive in silico analysis reveals a potential linkage between gene expression and hormone signaling that stimulates plant growth and metabolism
Source: PeerJ. 2020 Nov 4;8:e10053. doi: 10.7717/peerj.10053 (PMC7648454; doi:10.7717/peerj.10053)
Supplement: Supplemental Information 6 [file peerj-08-10053-s006.docx]

**Silicon flow from root to shoot in pepper: A comprehensive *in silico* analysis reveals a potential linkage between gene expression and hormone signaling that stimulates plant growth and metabolism**

Fernando Carlos Gómez-Merino*, Libia Iris Trejo-Téllez, Atonaltzin García-Jiménez, Hugo Fernando Escobar-Sepúlveda, and Sara Monzerrat Ramírez-Olvera

**Supplemental File SF5**

List of *cis*-regulatory elements responsive to phytohormones found in the promoter regions of *Lsi1* and *Lsi2* genes in the pepper (*Capsicum annuum*) genome.

| ***Cis*-acting element** | **Consensus sequence** | **Function** | **Genes containing the *cis*-acting element in their corresponding promoters** |
| --- | --- | --- | --- |
| **Abscisic acid responsive element** | | | |
| ABRE | ACGTG  CACGTA  CACGTG  CGCACGTGTC  TACGGTC  TACGTG  AACCCGG | Abscisic Acid Responsive Element, *cis*-acting elements involved in the abscisic acid responsiveness | *CaLsi1.1*, *CaLsi1.2*, *CaLsi1.4*, *CaLsi1.5*, *CaLsi1.7*, *CaLsi1.11*, *CaLsi1.12* and *CaLsi1.16* |
| G-Box | CACGTC  CACGTG  CACGTT  CACGAC  TAAACGTG  TACGTG | *cis*-acting involved in response to abscisic acid, methyl-jasmonate, ethylene, anaerobiosis and light | *CaLsi1.2*, *CaLsi1.4*, *CaLsi1.5*, *CaLsi1.7*, *CaLsi1.9, CaLsi1.11*, *CaLsi1,12*, and *CaLsi1.16* |
| **Jasmonic acid** | | | |
| CGTCA-motif | CGTCA | *cis*-acting regulatory element involved in the methyl jasmonate-responsiveness | *CaLsi1.2*, *CaLsi1.7*, *CaLsi1.9*, *CaLsi1.11*, *CaLsi12*, *CaLsi1.16, CaLsi2.1,* and *CaLsi2.3* |
| TGACG-motif | TGACG  AACGAC | *cis*-acting regulatory element involved in the methyl jasmonate-responsiveness | *CaLsi1.2*, *CaLsi1.7*, *CaLsi1.7*, *CaLsi1.9*, *CaLsi1.11*, *CaLsi1.12, CaLsi1.16, CaLsi2.1,* and *CaLsi2.3* |
| **Ethylene** | | | |
| ERE | ATTTTAAA | Ethylene Responsive Element, *cis*-acting regulatory element involved in the ethylene-responsiveness | *CaLsi1.1*, *CaLsi1.4*, *CaLsi1.5*, *CaLsi1.7*, *CaLsi1.9*, *CaLsi1.10,* and *CaLsi1.16* |
| **Salicylic acid** | | | |
| TCA-element | TCATCTTCAT  CCATCTTTTT  TCAGAAGAGG | *cis*-acting element involved in salicylic acid responsiveness | *CaLsi1.2*, *CaLsi1.4*, *CaLsi1.5* and *CaLsi1.9* |
| **Auxin** | | | |
| TGA-element | AACGAC | *cis*-acting element involved in auxin responsiveness | *CaLsi1.2*, *CaLsi1.12,* *CaLsi1.16,* and *CaLsi2.3* |
| AuxRR-core | GGTCCAT | Auxin Regulatory Responsive element, *cis*-acting element involved in auxin responsiveness | *CaLsi1.4* and *CaLsi1.5* |
| **Gibberellic acid** | | | |
| GARE-motif | CCTTTTG  TCTGTTG | Gibberellic Acid Response Element, *cis*-acting element involved in gibberellin responsiveness | *CaLsi1.7*, *CaLsi1.11, CaLsi2.1,* and *CaLsi2.3* |
| P-Box | CCTTTTG | *cis*-acting element involved in gibberellin responsiveness | *CaLsi1.7*, *CaLsi1.11,* *CaLsi1.16*, *CaLsi2.1*, and *CaLsi2.3* |
| TATC-Box | TATCCCA | *cis*-acting element involved in gibberellin responsiveness | *CaLsi2.1* |
