# Supplementary material for: Silicon flow from root to shoot in pepper: a comprehensive in silico analysis reveals a potential linkage between gene expression and hormone signaling that stimulates plant growth and metabolism
Source: PeerJ. 2020 Nov 4;8:e10053. doi: 10.7717/peerj.10053 (PMC7648454; doi:10.7717/peerj.10053)
Supplement: Supplemental Information 7 [file peerj-08-10053-s007.docx]

**Silicon flow from root to shoot in pepper: A comprehensive *in silico* analysis reveals a potential linkage between gene expression and hormone signaling that stimulates plant growth and metabolism**

Fernando Carlos Gómez-Merino*, Libia Iris Trejo-Téllez, Atonaltzin García-Jiménez, Hugo Fernando Escobar-Sepúlveda, and Sara Monzerrat Ramírez-Olvera

**Supplemental File SF6**

List and characteristics of Si-regulated proteins reported by Manivannan *et al*. (2016) in pepper (*Capsicum annuum*)*.*

| **UniProtKB accession** | **Gene Locus** | **Length (aa)** | **Molecular mass (kDa)** | **Protein name** | **Species** | **EST** |
| --- | --- | --- | --- | --- | --- | --- |
| A0A1U8FMW4 | XP_016560597 | 509 | 55.659 | Adenylosuccinate synthetase, chloroplastic | *Capsicum annum* | XM_016705110.1 |
| A0A2G3AID8 | XP_016544745 | 453 | 50.856 | E3 ubiquitin-protein ligase PUB23-like | *Capsicum annum* | NM_001324634.1 |
| Q71AU8 | AAQ10954 | 261 | 27.982 | Zinc finger protein | *Capsicum annum* | AF539746.1 |
| A0A1U8EKZ4 | XM_016692387 | 673 | 76.297 | Vacuolar protein sorting-associated protein 53 A-like isoform X2 | *Capsicum annum* | XM_016692396.1 |
| A0A1U8QD66 | YP_006666038 | 477 | 52.920 | Ribulose bisphosphate carboxylase large chain | *Capsicum annum* | YP_006666038.1 |
| A0A2G2ZWG5 | XP_016565613 | 492 | 55.855 | tRNA-specific 2-thiouridylase mnmA | *Capsicum annum* | XM_016710127.1 |
| A0A2G2ZUX2 | XP_016566587 | 376 | 42.544 | GDP-mannose 3,5-epimerase 2 | *Capsicum annum* | XM_016711101.1 |
| A0A1U8EVQ8 | XP_016551243 | 381 | 42.096 | Nucleoporin | *Capsicum annum* | XM_016695757.1 |
| A0A1U8FSU6 | XP_016562222 | 173 | 19.507 | Calcium-binding protein CML19 | *Capsicum annum* | XM_016706736.1 |
| Q6RWD8 | XP_016560735 | 180 | 20.392 | Ribulose bisphosphate carboxylase small chain | *Capsicum annum* | XP_016560735.1 |
| A0A1U8F4Z2 | XP_016554457 | 907 | 103.500 | Transcriptional activator DEMETER-like isoform X2 | *Capsicum annum* | XM_016698971.1 |
| A0A1U8FLS3 | XP_016557283 | 128 | 13.414 | Oxygen-evolving enhancer protein 3, chloroplastic-like isoform X1 | *Capsicum annum* | XM_016701797.1 |
| A0A2G2ZD87 | XP_016578025 | 194 | 21.532 | Molybdopterin synthase catalytic subunit | *Capsicum annum* | XM_016722539.1 |
| A0A1U8DWI0 | XP_016539377 | 234 | 26.698 | MADS-box transcription factor 23-like isoform X2 | *Capsicum annuum* | XM_016683891.1 |
| A0A1U8E9L6 | XP_016543897 | 1601 | 183.383 | Disease resistance protein RPS2-like isoform X1 | *Capsicum annum* | XM_016688411.1 |
| A0A1U8E1L2 | XP_016541163 | 222 | 25.687 | Mediator of RNA polymerase II transcription subunit 20 | *Capsicum annum* | XM_016685677.1 |
| J7H6Z8 | YP_006666067 | 134 | 15.240 | 50S ribosomal protein L16, chloroplastic | *Capsicum annum* | NC_018552.1 |
| G0Y2N6 | AEJ73201 | 45 | 5.182 | CIN-like protein, partial | *Nandina domestica* | AY212941.1 |
| A0A1S3ZVV1 | XP_016468560 | 294 | 33.764 | Calcium uniporter protein 5, mitochondrial-like isoform X1 | *Nicotiana tabacum* | XM_016613074.1 |
| A0A1U8FE34 | XP_016554559 | 1356 | 153.502 | Putative disease resistance RPP13-like protein 1 | *Capsicum annum* | XM_016699073.1 |
| Q1I0X5 | NP_001311913 | 511 | 55.409 | Pyruvate kinase | *Capsicum annum* | NM_001324984.1 |
| A0A1U8E4Y4 | XP_016541481 | 272 | 30.494 | Putative receptor-like protein kinase | *Capsicum annum* | XM_016685995.1 |
| A0A1U8HDS2 | XP_016581536 | 401 | 42.381 | Phosphoglycerate kinase | *Capsicum annum* | XM_016726050.1 |
| Q84X59 | AAO40761 | 279 | 30.920 | Ve Resistance gene-like protein | *Solanum tuberosum* | AY212941.1 |
| Q8RUZ2 | AF348370_1 | 104 | 10.935 | Beta-keto acyl reductase | *Zea mays* | AF348370.1 |
| Q8SA81 | AF467245_1 | 283 | 31.338 | Ve resistance gene analog | *Solanum tuberosum* | AF467245.1 |
| J7H3P2 | YP_006666015 | 507 | 55.492 | ATP synthase CF1 alpha subunit (chloroplast) | *Capsicum annum* | NC_018552.1 |
| Q711G5 | CAC87838.1 | 224 | 26.906 | Cullin 1D | *Nicotiana tabacum* | AJ344536.1 |
| A0A1U8F8L5 | XP_016552584 | 155 | 18.013 | F-box only protein 8-like | *Capsicum annum* | XM_016697098.1 |
| A0A2G2ZKT6 | XP_016573914 | 511 | 55.832 | Double-stranded RNA-binding protein 2-like | *Capsicum annuum* | XM_016718428.1 |
| UPI00051BAD89 | XP_009588623 | 192 | 22.053 | Uncharacterized protein LOC104086136 | *Nicotiana tomentosiformis* | XM_009590328.2 |
| A0A1U8FKW3 | PHT93852 | 438 | 50.233 | CBL-interacting protein kinase 33 | *Capsicum annuum* | AYRZ02000001.1 |
| 0A1U8HAZ6 | XP_016577584 | 314 | 35.680 | Calcium uniporter protein 6, mitochondrial-like isoform X2 | *Capsicum annuum* | XM_016722098.1 |
| K7QG79 | AFS89519 | 91 | 10.727 | Reverse transcriptase, partial | *Capsicum annuum* | JQ026398.1 |
| A0A1U8F5V2 | NP_001311631 | 202 | 23.037 | Eukaryotic translation initiation factor | *Capsicum annum* | NM_001324702.1 |
| Q8S9H3 | AAL76090 | 150 | 16.255 | Minichromosome maintenance protein 3, partial | *Nicotiana tabacum* | AY077639.1 |
| A0A0D5NRV2 | NP_001311850 | 453 | 48.319 | Glyceraldehyde-3-phosphate dehydrogenase | *Capsicum annum* | NM_001324921.1 |
| B5LAT9 | NP_001311511 | 247 | 27.823 | Caffeoyl-CoA O-methyltransferase 6 | *Capsicum annum* | NM_001324582.1 |
| A0A1U8EK48 | XP_016544254 | 244 | 27.373 | Ras-related protein RABH1b isoform X1 | *Capsicum annum* | XM_016688768.1 |
| A0A1U8GF17 | XP_016567323 | 387 | 44.090 | F-box/kelch-repeat protein At3g23880-like | *Capsicum annum* | XM_016711837.1 |
